# Evans–Polanyi-like Formulations for Rapidly Predicting Key Depolymerization Barriers in Xylopyranoses: Toward the Faster Development of Kinetic Models for Hemicellulose Pyrolysis

**DOI:** 10.1021/acs.jpca.5c00675

**Published:** 2025-05-14

**Authors:** Leandro Ayarde-Henríquez, Jacopo Lupi, Bernardo Ballotta, Stephen Dooley

**Affiliations:** † School of Physics, 8809 Trinity College Dublin, Dublin 2 D02 PN40, Ireland; ‡ AMBER, Advanced Materials and BioEngineering Research Centre, Dublin 2 D02 PN40, Ireland; § CNR-ICCOM, Consiglio Nazionale delle Ricerche, Via Giuseppe Moruzzi 1, Pisa I-56124, Italy

## Abstract

This work elucidates Evans–Polanyi-like (EPL) relations to rapidly estimate the standard activation enthalpy of three ubiquitous reaction classes playing a central role in hemicellulose pyrolysis: ring-opening, ring contraction, and elimination. These models bypass computing the reaction enthalpy by leveraging computationally cheap local and global electron-density-based chemical reactivity descriptors, such as Fukui’s functions (*f*), electron population of C–O bonds (*N*), and the gross intrinsic strength bond index (Δ*g* ^pair^), evaluated for reactants solely. More than 270 reactions observed in twenty-eight functionalized β-d-xylopyranoses, the hemicellulose building block, are used under the 20–80% partition scheme for validating-deriving purposes. By using multilinear regression analysis, four EPL equations are proposed for informing barriers at the M06–2X/6–311++G­(d,p), CBS-QB3, G4, and DLPNO–CCSD­(T)-F12/cc-pVTZ-F12//M06–2X/6–311++G­(d,p) levels. An adjusted coefficient of determination of 0.80 characterizes these parametric polynomials. Moreover, MAE and RMSE of ≈3.3 and ≈4.1 kcal mol^–1^ describe the performance of the best-fitting models at DFT and G4. Conversely, the highest values, MAE = 3.6 and RMSE = 4.7 kcal mol^–1^, are associated with the CBS-QB3 level. The benchmarking of the computed activation enthalpies at 298 K yields simple functions for high-level estimations from low levels of theory with *R* ^2^ ranging from 0.94 to 0.98. Extrapolating the DPLNO barriers to the complete basis set limit tends to lower them by 0.63 kcal mol^–1^. EPL expressions are tailored to facilitate the development of chemical kinetic models for hemicellulose pyrolysis, as the reactant structure is the only input required.

## Graphical abstract

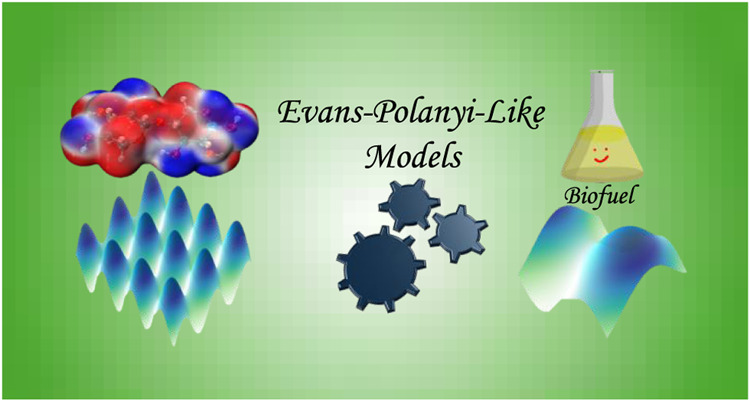

## 1 Introduction

### 1.1 Biomass Valorization

Biomass conversion to chemicals and fuels has captured the interest of industry and academia,^1,2^ as this matrix of organic matter is a plentiful and renewable reservoir of carbon and hydrogen with the potential to provide up to 147 exajoules in 2030,^3,4^ representing about 36% of the actual energy consumption.^3,4^ Plant biomass comprises diverse compounds, including lipids, sugars, and lignocellulose (building block). Lignocellulosic matter is mainly composed of cellulose (40–60%), hemicellulose (15–30%), and lignin (10–25%).^1,5^ Cellulose is the most abundant saccharide on Earth,^6,7^ showing an ordered polymeric structure of glucose (C_6_H_12_O_6_). In contrast, hemicellulose is not a homopolysaccharide as it is made of shorter chains, primarily hexoses (C_6_H_12_O_6_) and pentoses (C_5_H_10_O_5_).^8,9^ Conversely, lignin is an amorphous polymeric structure of aromatic ether rings attached to a three-carbon propene tail.^10–12^ Currently, the bio-oil produced falls short in quality and cost-effectiveness compared to its petroleum-derived crude counterparts due to several issues,^1,3,13^ including the need for predictive and unified kinetic models.

### 1.2 Chemical Kinetic Models for Hemicellulose Pyrolysis

Understanding hemicellulose pyrolysis has progressed through chemical kinetic models.^14–18^ While these models primarily focused on cellulose depolymerization, they have been applied to investigate hemicellulose decomposition. The seminal works of Broido^19,20^ and Shafizadeh^21,22^ introduced the classical architecture for cellulose pyrolysis. This three-reaction network considers cellulose to transform into active cellulose (intermediate) irreversibly. Each process is modeled by a first-order Arrhenius expression with the pre-exponent and the activation energy fitted to replicate TGA-derived data. Ranzi and co-workers^16–18^ proposed a sophisticated model for hemicellulose pyrolysis, including intermediates and pseudospecies, and defined stoichiometric coefficients for products. Broadbelt et al.^23^ extended this understanding by developing a detailed mechanistic model comprising 504 reactions and 114 species for fast pyrolysis of hemicellulose extracted from corn stover. The significant information requirements of this approach stress its data-intensive nature. Additionally, existing models are approximate in detail,^1,24^ mirroring the need for efficient tools to rapidly estimate thermodynamic properties for developing more robust architectures. This step is crucial considering the actual experimental limitations.

### 1.3 Methods for Barriers Estimation

Activation enthalpies are frequently challenging to determine experimentally or computationally. Density functional theory (DFT)^25^ functionals and post-Hartree–Fock (e.g., CCSD­(T)) methods are commonly used for exploring potential energy surfaces (PESs). Although CCSD­(T) is recognized for predicting barriers within chemical accuracy (±1 kcal mol^–1^), alternative tools such as machine learning-driven algorithms have been developed to reduce computational costs. Typically, DFT and CCSD­(*T*) show scaling of *O*(*N*_0_^3–4^) and *O*(*N*_0_^7^), respectively, with *N*_0_ representing the number of electrons. Similarly, the Evans–Polanyi (EP) principle,^26–28^ also referred to as Bell-Evans–Polanyi^29^ and Brønsted-Evans–Polanyi,^30,31^ is a basic rule establishing an empirical linear relation between the activation energy, *E*_a_, and the corresponding change in the reaction enthalpy, Δ*H*_r_ for elementary chemical reactions with similar transition states (TSs). By leveraging more accessible thermodynamic data (i.e., enthalpy of formation) of reactant(s) and product(s), the EP relation bypasses the TS complexities. Because of these advantages, this model has been extensively applied across several chemistries, including substitutions,^27^ photoreactions,^32^ hydrogen transfers,^33^ electrocatalysis,^34^ surface reactions on transition metal^35,36^ and transition metal oxides,^37,38^ and hydrocarbon^39^ and synthetic/natural polymeric biomass pyrolysis.^40^ This versatility highlights the potential of simple fundamental principles to explore PESs efficiently, making the EP expression particularly attractive.

### 1.4 Electron-Density-Based Chemical Reactivity Prediction

Chemical reactions are rationalized in terms of electron rearrangements in the Born–Oppenheimer approximation.^41^ Such charge reorganizations are the driving force of the bond-breaking and bond-forming processes, key components of reaction mechanisms. A reaction mechanism is a sequence of elementary steps detailing the reactant(s) chemical transformations to product(s). However, the chemical bond is a nonphysical observable, as asserted by Coulson.^42^ Fortunately, the Hohenberg–Kohn theorems^25^ establish that the electron density, *ρ*(**r**), determines the ground-state properties of systems. This local function is defined within the many-body theory^43–45^ and can be experimentally derived from Bragg intensities using X-ray diffraction techniques,^46,47^ in contrast to the wave function. Various density-based approaches have been developed to investigate the molecular systems’ chemical reactivity, including conceptual density functional theory (CDFT),^48^ quantum chemical topology (QCT),^49^ and interaction strength schemes.^50,51^

#### 1.4.1 Fukui’s Functions

CDFT applies Hohenberg–Kohn’s theorems^25^ to derive local and global response functions.^52,53^ These indices have enabled rigorous redefinitions of key chemical concepts such as electronegativity and aromaticity have been redefined rigorously, thereby allowing a fundamental understanding of reaction mechanisms and enabling their control.^54,55^ Fukui’s functions^56^ are CDFT local descriptors broadly applied to gain insights into radical^57^ and ring-closure^58^ processes, and the derived selectivity learnings have been used in drug design and material discovery fields,^59,60^ biomass pyrolysis,^61,62^ and biomass materials.^63–65^

#### 1.4.2 Bonding Analysis Based on the Electron Localization Function (ELF)

QCT extracts chemical information from the topological analysis of scalar functions’ gradient vector field. These approaches define topological domains via parameter-free partitioning schemes, as the reference state is not explicitly mentioned.^66^ Within QCT, ELF bonding analysis is an appealing interpretative framework allowing the recovery of Lewis empirical notions such as electron sharing, core, and lone pairs. ELF was elucidated within Hartree–Fock’s formalism as a quantum probe for visualizing Pauli’s exclusion principle and has notably been derived from X-ray diffraction data.^67,68^ This ELF-based approach has been applied from the gas phase to the solid state, enriching our understanding of ubiquitous reaction mechanisms at the electronic level, including hemiaminals formation,^69^ different covalent bonding situations,^70,71^ cycloadditions,^72–78^ phase transition of the 14 group elements,^79^ photoreactions,^80–84^ arynes production,^85^ deducing linear models for predicting activation enthalpies of organic and organometallic systems,^86^ and computing local and global properties of solids.^87^

#### 1.4.3 Intrinsic Bond Strength Index (IBSI)

The independent gradient model (IGM)^88^ leverages ∇*ρ*(**r**) topographical analysis to distinguish chemical bonds’ intensity via the intrinsic bond strength index (IBSI).^89^ IBSI has proven useful in studying various molecular systems, including hydrogen-bonded,^89^ halogen-bonded,^90^ organometallics for drug design^91^ and optical materials discovery,^92^ metal coordination,^89^ and stabilizing bioproducts for aerosol formation.^93^ These applications have demonstrated IBSI’s hierarchy, with higher values observed in covalent bonds, intermediate for transition metal coordination bonds, and the lowest for weak interactions, underscoring IBSI’s efficacy in quantitatively classifying chemical interactions’ strength. In this work, however, the unnormalized form of IBSI (i.e., excluding the H_2_ normalization) has been utilized as it led to better fits. Note that no fundamental physics is lost using the gross instead of normalized values.

### 1.5 Work’s Goals

This work aims to derive EP-like (EPL) relations to rapidly estimate the activation enthalpies of three ubiquitous reaction classes central in organic synthesis,^94,95^ pharmaceutical chemistry,^96,97^ and biomass pyrolysis:^98–104^ ring-opening, ring contraction, and elimination. Over the past two decades, extensive studies have integrated these thermal-induced processes into kinetic models to describe the formation and evolution of products resulting from the depolymerization of β-d-xylopyranose (hereafter termed xylose).^98–104^ Herein, we applied and validated the EPL polynomials within the promising hemicellulose pyrolysis field, using xylose (the most abundant monosaccharide in hemicellulose^105^) as the structural motif and gauging Fukui’s functions, electron population derived from the ELF bonding analysis, and gross IBSI. Poor results were observed when applying the original EP relation. Indeed, various chemical reactivity descriptors are available in the literature; however, the combination of the chosen indices produced the best predictive models. Indeed, developing chemical kinetic models requires additional thermodynamic data, including enthalpy of formation and activation entropy. This information can be readily computed using the methodologies described in refs 106,107 .

The manuscript is organized as follows: First, the computational methods and theoretical foundations are outlined. Subsequently, the performance of the EPL relations is discussed. The last section summarizes the main findings and suggests directions for future research.

## 2 Methods and Theory

### 2.1 Electronic Methods

Geometry optimizations and frequency calculations were performed using global hybrid functionals, including M06–2X, ωB97X-D, and Becke three-parameter Lee–Yang–Parr (B3LYP). The first combined with the 6–311++G­(d,p) Pople’s split-valence basis set,^108^ while the other two long-range corrected functionals with the smaller 6–31+G­(d,p).^108^ These calculations were conducted utilizing the Gaussian 16 code.^109^ M06–2X is parametrized for nonmetal elements and has been recommended for main-group thermochemistry.^110^ Similarly, ωB97X-D reoptimization includes empirical atom–atom dispersion corrections, making it suitable for thermochemistry and kinetics.^111^ B3LYP^112–114^ is a popular functional utilized broadly to investigate reaction mechanisms in the gas phase. For all cases, the intrinsic reaction coordinate (IRC)^115^ was traversed to ensure the TS connects the correct reactant and product(s). As a standard procedure, the stationary points on the IRC were characterized as minima (i.e., reactants, intermediates, and products) and first-order saddle points (TSs) based on the vibrational analysis. The adequacy of our single-reference-based methodology was assessed via the $T_{1}$ diagnostic^116^ for key species along the pathways. The static correlation is insignificant for the investigated systems, meaning multireference methods are not required since $T_{1}$ < 0.02. See the Supporting Information (SI) for further details.

Composite model chemistry methods such as the complete basis set (CBS-QB3)^117,118^ and Gaussian-4 (G4)^119^ were employed to obtain accurate thermochemistry values at both absolute zero and 298 K. In these approaches, geometry optimizations and frequency calculations are performed using relatively simple and cost-effective methods, adding higher levels of theory stepwise. Gaussian 16 was utilized for calculations involving these two composite methods. Additionally, the domain-based local pair natural orbital (DLPNO),^120,121^ used to accelerate the canonical CCSD­(T), was combined with the *de facto* F12 geminal functions^122^ and the correlation-consistent cc-pVTZ basis set.^123,124^ This explicit correlated method was employed for single-point calculations on preoptimized geometry at M06–2X/6–311++G­(d,p). The TightPNO setting was used to achieve high accuracy with a sharp error distribution,^125^ serving the cc-pVQZ basis set as the complementary auxiliary basis set (CABS) for F12 methods.^126^ The ORCA 5.0 ^126^ package of programs was utilized to compute the barriers at DLPNO–CCSD­(T)-F12/cc-pVTZ-F12 within the frozen core approximation.^127^ These high-correlated electronic energies were finally combined with thermodynamic corrections derived from frequencies at the M06–2X/6–311++G­(d,p) level to compute the activation enthalpies at 298 K.^128^ The treatment of anharmonicities was integrated into the analysis by considering a scaling factor of 0.970.^129^ This was conducted with the Shermo suite.^130^ The extrapolation to the complete basis set (CBS) limit using both cc-pVDZ and cc-pTVZ was considered to correct for basis-set truncation errors. This was achieved via the two-point extrapolation scheme using the ORCA matrix-driven module where the SCF, *E*_SCF_^∞^, and correlation, *E*_corr_^∞^, energies at the basis set limit are determined by the following expressions^131^

1 $$E_{\mathrm{SCF}}^{X}=E_{\mathrm{SCF}}^{\infty }+Ae^{-\alpha \sqrt{X}}$$

2 $$E_{\mathrm{corr}}^{\infty }=\frac{X^{\beta }E_{\mathrm{corr}}^{X}-Y^{\beta }E_{\mathrm{corr}}^{Y}}{X^{\beta }-Y^{\beta }}$$

where *A* is a number to be determined for each system, *α* and *β* are optimized parameters for basis-set pairs (in this study, *α* = 4.42 and *β* = 2.46 ^131^), and *Y* and *X* represent cardinal numbers equal to 2 and 3, respectively.

### 2.2 Evaluation of Chemical Reactivity Descriptors

EPL models incorporate density-based chemical reactivity indices as independent variables, including Fukui’s functions,^56^ *f* ^+^, *f* ^–^, and *f* ^0^, of carbon (C) and oxygen (O) atoms, the electron population, *N*, of C–O and C–C bonds, and the unnormalized IBSI,^89^ Δ*g* ^pair^, i.e., the integrated form of a local chemical reactivity index, δ*g*, defined in the IGM framework and computed for C–C and C–O. The numerical evaluation of these functions was carried out using the Multiwfn packages.^132^ For the *f* indices, the default grid was utilized as these descriptors have a low-grid dependency, meaning that 32,550 points were needed. For the topographical analysis of ELF, however, the high-quality grid was used to obtain accurate integrated values; thus, for xylose (the smallest system, i.e., 80e), the total number of points was found to be 42,836,904, whereas for glucuronoxylan (the bulkiest system, i.e., 180e) 79,242,210. Similarly, Δ*g* ^pair^ was computed using the ultrafine-quality grid, signifying that 18,120 points were required for all examined species. Increasing the quality of the integration grid further led to almost the same values, underscoring the computational cost-effectiveness of this method.

#### 2.2.1 Fukui’s Functions

The *f* indices characterize the local response of the system to nucleophilic (*f* ^+^), electrophilic (*f* ^–^), and neutral/radical (*f* ^0^) attacks.^56,133^ The higher the value of these descriptors at a particular site, the more reactive this region is relative to others, and in the canonical ensemble at absolute zero, one gets the following finite-difference-type expressions^52,53^

3 $$f^{+}\left(r\right)={\left(\frac{\partial \rho \left(r\right)}{\partial N_{0}}\right)}_{v}^{+}=\rho_{N_{0}+1}\left(r\right)-\rho_{N_{0}}\left(r\right)$$

4 $$f^{-}\left(r\right)={\left(\frac{\partial \rho \left(r\right)}{\partial N_{0}}\right)}_{v}^{-}=\rho_{N_{0}}\left(r\right)-\rho_{N_{0}-1}\left(r\right)$$

5 $$f^{0}\left(r\right)=\frac{\rho_{N_{0}+1}\left(r\right)+\rho_{N_{0}-1}\left(r\right)}{2}$$

where **r** is the position vector, and *v*(**r**) represents the external potential (typically produced by nuclei). However, eqs 3–5 need to be rewritten in the so-called condensed-to-atom fashion to extract quantitative information at the atomic level^133,134^

6 $$f_{A}^{+}=N_{N_{0}+1}^{A}-N_{N_{0}}^{A}$$

7 $$f_{A}^{-}=N_{N_{0}}^{A}-N_{N_{0}-1}^{A}$$

8 $$f_{A}^{0}=\frac{N_{N_{0}+1}^{A}+N_{N_{0}-1}^{A}}{2}$$

In eqs 6–8, *N*_*N*_0__^A^, *N*_*N*_0_–1_^A^, and *N*_*N*_0_+1_^A^ correspond to the electron population of atom A in its neutral, cationic, and anionic states, respectively. Although several population analyses can be used to compute this quantity (e.g., Mulliken^135^ and Bader^136^ schemes), we here adopted the Hirshfeld^137^ partition scheme as it has been shown to be an adequate method to bridge the gap between experimental observations and computational-derived numbers in various chemical reactivity scenarios.^138^ Moreover, Roy et al.^139^ showed that this framework stabilizes the Fukui’s functions value by lowering their probability of becoming negative.

#### 2.2.2 ELF’s Topographical Analysis

The electron population of C–C and C–O was derived from the topographical analysis of ELF, *η*(**r**)^140^

9 $$0\leq \eta \left(r\right)=\frac{1}{1+{\left(\tau_{P}\left(r\right)/\tau_{H}\left(r\right)\right)}^{2}}\leq 1$$

10 $$\tau_{P}\left(r\right)=\frac{1}{2}\sum_{i}^{N_{0}}{\left|\nabla \psi_{i}\left(r\right)\right|}^{2}-\frac{1}{8}\frac{{\left|\nabla \rho \left(r\right)\right|}^{2}}{\rho \left(r\right)}$$

11 $$\tau_{H}\left(r\right)=\frac{3}{5}{\left(6\pi^{2}\right)}^{2/3}\rho {\left(r\right)}^{5/3}$$

where *ψ*_*i*_(**r**) represent the Hartree–Fock (or Kohn–Sham) molecular orbitals, *τ*_P_(**r**) and *τ*_H_(**r**) are the local positive-definite Pauli and Thomas-Fermi kinetic energy densities, respectively. ELF is frequently interpreted as a local measure of electrons’ kinetic energy excess due to Pauli’s exclusion principle;^141^ thus, high values of this function (>0.5) correspond to spatial positions **r** with high electron localization.^142,143^ The nullity condition applied to the ELF gradient, ∇*η*(**r**) = 0, yields a partition of the molecular space into basins of attractors empirically associated with Lewis’ bonding elements, including valence bonds, lone pairs, and atomic cores.^144–146^ Notably, these regions are located in almost the same position that the valence-shell electron pair repulsion (VSEPR)^147^ theory predicts.^144^ Moreover, such basins are typically described in terms of the synaptic order, which informs the number of core basins connected with a valence basin.^144^ Lone pairs are typical examples of valence monosynaptic basins, V­(A), while a two-center bond between atoms A and B is associated with a disynaptic basin, V­(A,B). The average electron population (i.e., expected value) of ELF basins can be computed by integrating *ρ*(**r**) over such domains, Ω

12 $$N=\left\langle \Psi \left(r\right)\left|\mathrm{N̂}\right|\Psi \left(r\right)\right\rangle =\int_{\Omega }\rho \left(r\right)dr$$

In eq 12, **N̂** is the quantum mechanical operator of the electron population (i.e., its eigenvalues), *N*, and Ψ­(**r**) is the *N*_0_-electron wave function. Thus, the eigenvalues of these operators linked to different basins are not independent, as evidenced by the closure relation^148^

13 $$\sum_{A}N=N_{0}$$

Several works have reported the notably high stability of ELF relative to the level of theory, meaning that the insights gained from ELF topographical analysis are almost invariant with respect to the method.^71,75,149,150^ Burdett and McCormick^150^ provided a theoretical argument to support the computational evidence. They demonstrated that ELF-derived quantities depend solely on the nodal properties of the occupied orbitals when the one-electron matrix adequately describes the system.

#### 2.2.3 Gross IBSI

Δ*g* ^pair^ is a global index measuring the tendency of electron density to be shared by a pair of interacting fragments (e.g., two atoms),^89^ and thus, it has been shown to correlate well with the notion of the chemical bond strength across different bonding situations.^89,151^ This quantity has been derived within the IGM^88^ framework to study both intra- and inter-fragment interactions through the δ*g*(**r**) function. The IGM-δ*g*(**r**) descriptor is a local measure of the electron charge shared, unveiling thus the molecular domains where the density *ρ*(**r**) accumulates. By gauging the topographical analysis of the ∇*ρ*(**r**) modulus resulting from the summation of interacting fragment densities in their free state, the following expression is derived for δ*g*(**r**)­

14 $$\delta g\left(r\right)=\sum_{A}\left|\nabla \rho_{A}^{\mathrm{IGM}}\left(r\right)\right|-\left|\sum_{A}\nabla \rho_{A}\left(r\right)\right|$$

where ∇*ρ* ^IGM^(**r**) and ∇*ρ*(**r**) are the gradient densities of the noninteracting reference and the associated real systems, respectively. Restricting the analysis to a pair of atoms leads to the δ*g* ^pair^(**r**) local descriptor, and its integration over the interaction domain Ω_*i*_ yields its global form, Δ*g* ^pair^

15 $$\Delta g^{\mathrm{pair}}=\int_{\Omega_{i}}\delta g^{\mathrm{pair}}\left(r\right)dr$$

Hénon and collaborators^89^ divided the values derived from eq 15 by the square of the distance of the atoms, *d*_AB_^2^, to properly compare different bond types. In this work, however, the evaluation of Δ*g* ^pair^ has been performed using a slightly different approach proposed by Lu and Chen.^152^ They replaced the free-state density of atoms with the actual ones to make the original framework more physical-chemical sounding and used the Hirshfeld scheme to compute such densities. The ultrafine-quality grid featuring 18,120 points was utilized for computing Δ*g* ^pair^. Extensive computational evidence indicates that this descriptor has low dependency on the level of theory used to obtain the electronic wave function.^89,152^

### 2.3 Implementation Details

The EP model^26–31^ straightforwardly incorporates key thermochemical properties into a simple mathematical expression

$$E_{a}=\epsilon \Delta H_{r}+\varepsilon$$ 16

where ϵ represents the slope (also referred to as the Brønsted coefficient) of the linear model and informs (roughly) about the TS position along the reaction pathway (e.g., a reactant- or product-like TS),^153^ and ε, the intercept of the equation, corresponds to the activation energy of a thermoneutral reaction, a hypothetical chemical process.^153^ Equation 16 can be rewritten in terms of the activation enthalpy, Δ*H* ^‡^, by resorting to the linear dependency between this quantity and *E*_a_ ^154^

$$\Delta H^{‡}=\varepsilon \Delta H_{r}+\varepsilon^{\prime }$$ 17

Note that the scaling procedure does not change the meaning of the slope and intercept. In quantum chemistry calculations, eq 17 is preferred over eq 16 as Δ*H* ^‡^ is the quantity obtained through electronic-based methodologies. The central idea of this work is to replace Δ*H*_r_ by a set containing chemical reactivity descriptors, including Fukui’s functions, the electron population, and the gross intrinsic bond strength index. These indices are to be computed for reactants exclusively, decreasing thereby the structures to be considered and speeding the algorithm

18 $$\Delta H_{r}\to {\left\{\mathrm{f̅},\mathrm{N̅},\overset{®}{\Delta g^{\mathrm{pair}}\;}\right\}}_{\mathrm{reactant}}$$

19 $$\mathrm{f̅}=\frac{\sum_{A}f_{A}^{0}+\sum_{B}f_{B}^{-}}{\sum A+\sum B}$$

20 $$\mathrm{N̅}=\frac{\sum_{i}V_{i}\left(\mathrm{A,B}\right)}{\sum i}$$

21 $$\overset{®}{\Delta g^{\mathrm{pair}}\;}=\frac{\sum_{A‐B}\Delta g_{A‐B}^{\mathrm{pair}}}{\sum A‐B}$$

Figure 1 shows the molecular graph of xylose (see Panel a), which has been derived from the topological analysis of the ELF gradient alongside its Lewis-like structure (see Panel b). The next Subsection defines the A and B atoms for each reaction class: ring-opening, ring contraction, and elimination; see Figure 1, Panel c. For simplicity, xylose is herein utilized as a prototype framework to elucidate the EPL models; thus, the approach discussed in this Subsection was methodically applied to the rest of the isomers.

**Figure 1:**
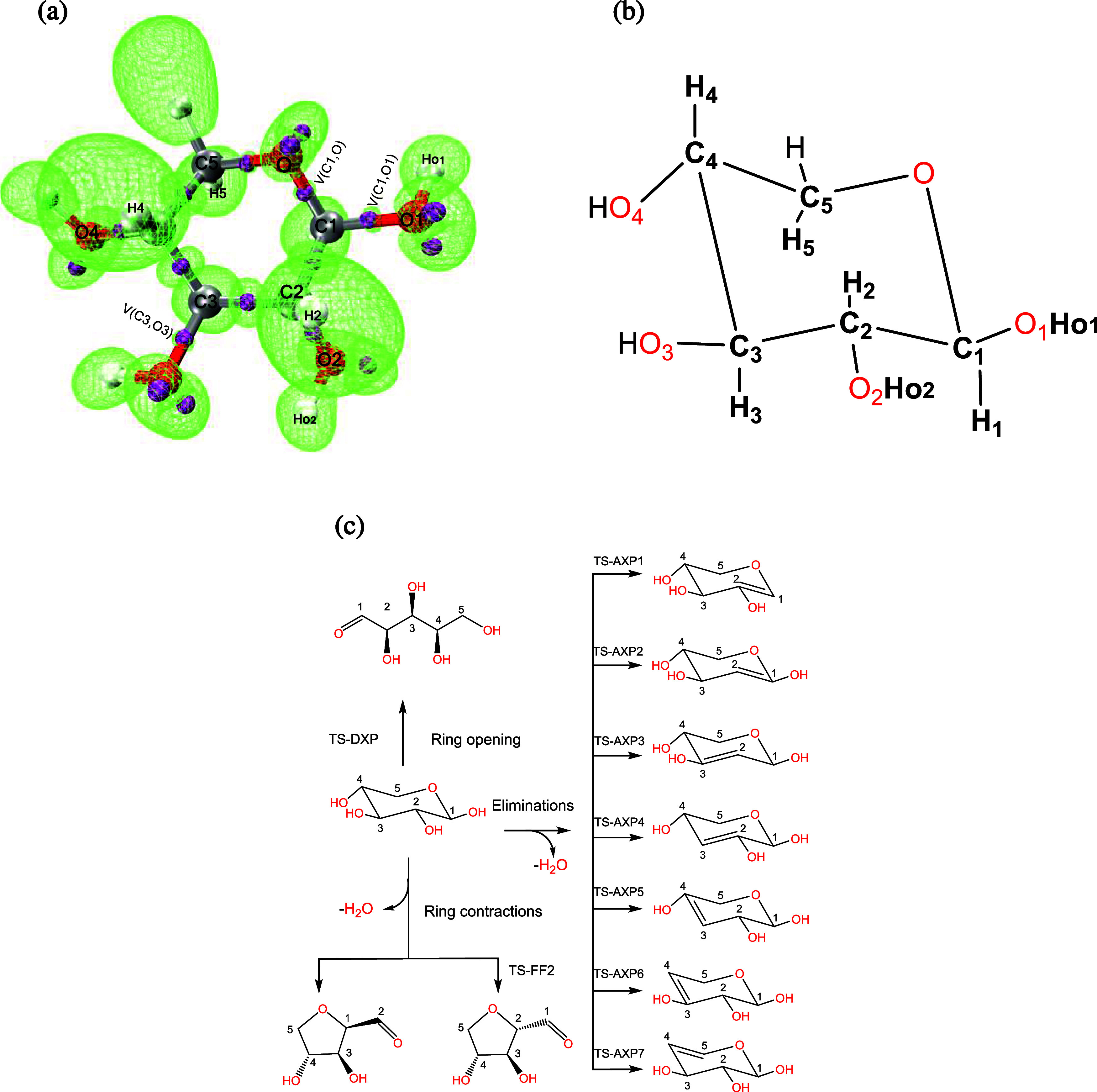
Xylose’s molecular graph (Panel a) altogether with its Lewis-like representation (Panel b). Panel c illustrates a kinetic model for the initial depolymerization steps of xylose: adapted with permission from ref 104. Copyright 2025 Elsevier. Some elements are color-coded in Panel a: green isosurfaces (*η* = 0.81) correspond to ELF basins, and purple spheres represent ELF attractors.

#### 2.3.1 The Ring-Opening Class

This elementary step leads to the acyclic product (DXP) through the bond scission between the pyran-ring oxygen (O) and the carbon at position 1 (C1) alongside the transfer of the proton bonded to O1 (H_O1_); see Figure 1, Panel c. The *f* ^0^ function was chosen for the C1 carbon, whereas *f* ^–^ for O and O1 oxygens. The arithmetic mean of this triad was the parameter included in the model. The electron population *N̅* of the disynaptic basin associated with the C1–O bond, V­(C1,O), was the second independent variable. Only valence basins of C–O bonds are considered for computing *N̅*. Lastly, Δ*g* ^pair^ was computed for C1–O and O1–H_O1_, and the arithmetic mean integrated into the model. This strategic approach allows accounting for all reacting centers involved in the process. Hence, the set elements considered in eqs 19–21 are defined in the following fashion for ring-opening

22 $$\mathrm{f̅}=\frac{f_{C1}^{0}+f_{O}^{-}+f_{\mathrm{O1}}^{-}}{3}$$

23 N̅=V(C1,O)

$$\overset{®}{\Delta g^{\mathrm{pair}}\;}=\frac{\Delta g_{C1-O}^{\mathrm{pair}}+\Delta g_{O1-H_{O1}}^{\mathrm{pair}}}{2}$$ 24

#### 2.3.2 The Ring Contraction Class

Furfural (FF) results from the ring contraction process through two channels. One, leading to FF1, involves the C2–C3 and C1–O1 scissions altogether with the proton (H_O2_) migration from O2 to O1; in contrast, the other process (producing FF2) comprises both C1–O and C2–O2 breakages, with H_O1_ bonding O2 (See Figure 1, Panel c). Following the methodology detailed in Subsection 2.3.1, the expressions ([Disp-formula eq25])–([Disp-formula eq27]) and ([Disp-formula eq28])–([Disp-formula eq30]) are defined for FF1 and FF2, respectively

25 $$\mathrm{f̅}=\frac{f_{C1}^{0}+f_{C2}^{0}+f_{C3}^{0}+f_{O1}^{-}+f_{O2}^{-}}{5}$$

26 N̅=V(C1,O1)

$$\overset{®}{\Delta g^{pair}\;}=\frac{\Delta g_{C2\text{−}C3}^{pair}+\Delta g_{C1\text{−}O1}^{pair}+\Delta g_{O2\text{−}H_{O2}}^{pair}}{3}$$ 27

28 $$\mathrm{f̅}=\frac{f_{C1}^{0}+f_{C2}^{0}+f_{O}^{-}+f_{O1}^{-}+f_{O2}^{-}}{5}$$

29 $$\mathrm{N̅}=\frac{V\left(C1\mathrm{,O}\right)+V\left(C2\mathrm{,O}2\right)}{2}$$

$$\overset{®}{\Delta g^{pair}\;}=\frac{\Delta g_{C1\text{−}O}^{pair}+\Delta g_{C2\text{−}O2}^{pair}+\Delta g_{O1\text{−}H_{O1}}^{pair}}{3}$$ 30

#### 2.3.3 The Elimination Class

Xylose undergoes eliminations at positions 1, 2, 3, and 4 upon the bond breakage of carbons at these sites and the oxygen atom of the hydroxyl group, producing anhydroxylopyranose (AXP) species (see Figure 1, Panel c). Similarly, this step encompasses a proton transfer from the neighboring carbon to the oxygen. As an illustrative example, AXP1 formation involves the C1, O1, and H2 centers

31 $$\mathrm{f̅}=\frac{f_{C1}^{0}+f_{C2}^{0}+f_{O1}^{-}}{3}$$

32 N̅=V(C1,O1)

$$\overset{®}{\Delta g^{pair}\;}=\frac{\Delta g_{C1\text{−}O1}^{pair}+\Delta g_{C2\text{−}H2}^{pair}}{2}$$ 33

The following relations provide a general way to evaluate the parameters required to describe the AXP_*i*_ (i∈N,1≤i≤7) formation via EPL relations

1.
$i=1\Rightarrow \mathrm{f̅}=\left\{f_{C_{i}}^{0},f_{C_{i}+1}^{0},f_{O_{i}}^{-}\right\},\mathrm{N̅}=\left\{V\left(C_{i},O_{i}\right)\right\},\overset{®}{\Delta g^{\mathrm{pair}}\;}=\left\{\Delta g_{C_{i}O_{i}}^{\mathrm{pair}},\Delta g_{C_{i+1}H_{i+1}}^{\mathrm{pair}}\right\}$

2.
$i=2\Rightarrow \mathrm{f̅}=\left\{f_{C_{i}}^{0},f_{C_{i-1}}^{0},f_{O_{i}}^{-}\right\},\mathrm{N̅}=\left\{V\left(C_{i},O_{i}\right)\right\},\overset{®}{\Delta g^{\mathrm{pair}}\;}=\left\{\Delta g_{C_{i}O_{i}}^{\mathrm{pair}},\Delta g_{C_{i-1}H_{i-1}}^{\mathrm{pair}}\right\}$

3.
$i=3\Rightarrow \mathrm{f̅}=\left\{f_{C_{i}}^{0},f_{C_{i-1}}^{0},f_{O_{i-1}}^{-}\right\},\mathrm{N̅}=\left\{V\left(C_{i-1},O_{i-1}\right)\right\},\overset{®}{\Delta g^{\mathrm{pair}}\;}=\left\{\Delta g_{C_{i-1}O_{i-1}}^{\mathrm{pair}},\Delta g_{C_{i}H_{i}}^{\mathrm{pair}}\right\}$

4.
$i=4\Rightarrow \mathrm{f̅}=\left\{f_{C_{i-1}}^{0},f_{C_{i-2}}^{0},f_{O_{i-1}}^{-}\right\},\mathrm{N̅}=\left\{V\left(C_{i-1},O_{i-1}\right)\right\},\overset{®}{\Delta g^{\mathrm{pai}r}\;}=\left\{\Delta g_{C_{i-1}O_{i-1}}^{\mathrm{pair}},\Delta g_{C_{i-2}H_{i-2}}^{\mathrm{pair}}\right\}$

5.
$i=5\Rightarrow \mathrm{f̅}=\left\{f_{C_{i-1}}^{0},f_{C_{i-2}}^{0},f_{O_{i-2}}^{-}\right\},\mathrm{N̅}=\left\{V\left(C_{i-2},O_{i-2}\right)\right\},\overset{®}{\Delta g^{\mathrm{pair}}\;}=\left\{\Delta g_{C_{i-2}O_{i-2}}^{\mathrm{pair}},\Delta g_{C_{i-1}H_{i-1}}^{\mathrm{pair}}\right\}$

6.
$i=6\Rightarrow \mathrm{f̅}=\left\{f_{C_{i-2}}^{0},f_{C_{i-3}}^{0},f_{O_{i-2}}^{-}\right\},\mathrm{N̅}=\left\{V\left(C_{i-2},O_{i-2}\right)\right\},\overset{®}{\Delta g^{\mathrm{pair}}\;}=\left\{\Delta g_{C_{i-2}O_{i-2}}^{\mathrm{pair}},\Delta g_{C_{i-2}H_{i-2}}^{\mathrm{pair}}\right\}$

7.
$i=7\Rightarrow \mathrm{f̅}=\left\{f_{C_{i-2}}^{0},f_{C_{i-3}}^{0},f_{O_{i-3}}^{-}\right\},\mathrm{N̅}=\left\{V\left(C_{i-3},O_{i-3}\right)\right\},\overset{®}{\Delta g^{\mathrm{pair}}\;}=\left\{\Delta g_{C_{i-3}O_{i-3}}^{\mathrm{pair}},\Delta g_{C_{i-2}H_{i-2}}^{\mathrm{pair}}\right\}$

#### 2.3.4 Algorithm’s Flowchart

Scheme 1 summarizes the algorithm underpinning the proposed methodology through a flowchart.

**Scheme 1:**
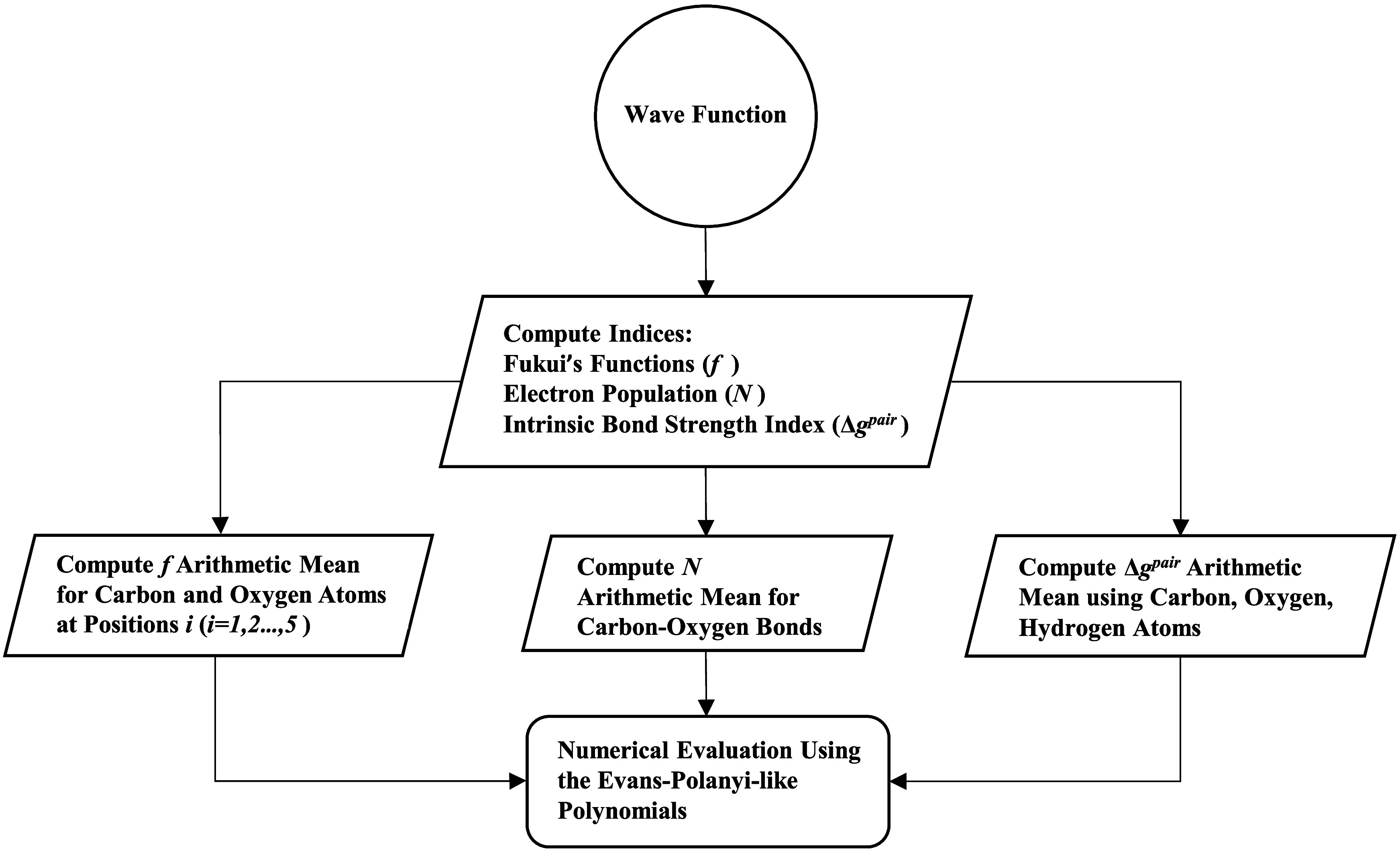
Simplified Flowchart of the Proposed Methodology, Showing the Algorithm for Estimating Standard Activation Enthalpies for Ring-Opening, Ring Contraction, and Elimination Reaction Classes

#### 2.3.5 Further Considerations and Refinements

The {*f*, *N*, Δ*g* ^pair^} collection has several advantages detailed in the Results and Discussion section: i. it is computationally cheap, ii. it yields accurate results, and iii. the geometry of reactants is the only structural variable the algorithm requires as input. However, in our experience, the algorithm can be fine-tuned further by keeping the value of Fukui’s functions of xylose’s reacting centers fixed, updating only these indices if they correspond to a site bearing a functional group. This procedure leads to a desired walltime reduction for computing activation enthalpies regarding the forthcoming algorithm coding while retaining the results’ accuracy.

#### 2.3.6 Assessment of the EPL Models Predictability

Statistical metrics such as the adjusted coefficient of determination, *R*_adj_^2^,^155^ the mean absolute error, MAE, and the root-mean-square error, RMSE, are used to evaluate the EPL models performance in predicting activation enthalpies. The need for multiple parameters stems from the insufficiency of any single descriptor to fully characterize the accuracy of regression models.^156^

## 3 Results and Discussion

### 3.1 Insights into the Features and Performance of the EPL Models

EPL relations leverage computationally cheap reactivity descriptors, i.e., Fukui’s functions, electron population of C–O bonds, and gross IBSI computed for reactants solely to rapidly estimate activation enthalpies of ubiquitous reaction classes playing a crucial role in hemicellulose pyrolysis:^98–104^ ring-opening, ring contraction, and elimination. By combining a gas-chromatograph (GC), coupled to a mass spectrometer (MS), and quantum chemistry methodologies, Lu and co-workers^104^ proposed a detailed kinetic model comprising 49 reactions. This network describes the intermediates and products forming from xylose’s decomposition through ring-opening, ring contraction, elimination, cyclization (acetal and hemiacetal), and isomerization (retro-aldol). Notably, the model accurately predicts the formation of five-membered rings, anhydroxylopyranose, glycolaldehyde, and dihydrofuran-3­(2*H*)-one. Commonly, xylose residues from hardwood contain approximately 70% of O-acetyl groups at positions 2 and/or 3 and 10% of both methoxy and carboxyl bonded at positions 4 and 5, respectively.^105^ The location sites of these groups have been determined by combining experimental techniques, such as nuclear magnetic resonance (NMR) and thermogravimetric analysis (TGA).^157–159^ Thus, the prominence of 2-*O*-acetyl, 4-methoxy, and 5-carboxyl have been considered in this study to model the molecular composition of hemicellulose plant matter accurately. Twenty-eight xylose-based species resulting from combining such functional groups are used to derive (80%) and validate (20%) the EPL models. Scheme 2, Panels b and c, depicts the reaction network detailing the initial depolymerization steps of two real-world functionalized species. The kinetic model of xylose is also included to facilitate comparison and better illustrate our methodology (see Scheme 2, Panel a). In these kinetic frameworks, ring-opening leads to an acyclic form, FF and FF-like (FFL) result from the ring contraction of xylose and its analogs, respectively, and elimination leads to AXP and AXP-like (AXPL) for unsubstituted and substituted systems, respectively.

**Scheme 2:**
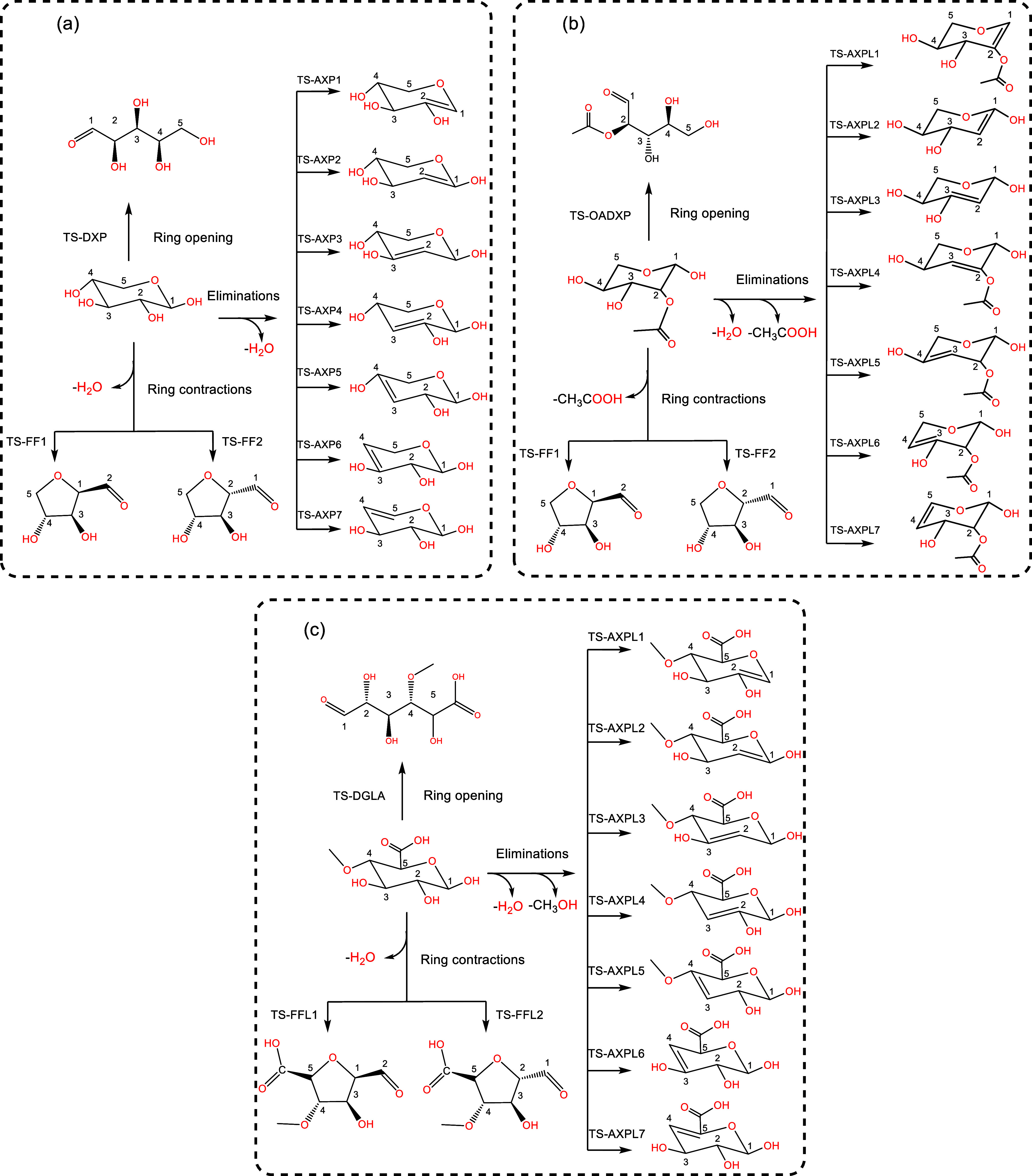
Lewis-like Structures Showing a Kinetic Model for the Initial Depolymerization of Xylose (Panel a^104^). All Twenty-Eight Functionalized Species, Including Mono- and Di-Saccharides, Undergo the Same Reactions. For Further Clarification, 2-*O*-Acetyl-xylose (Panel b) and 4-*O*-Methyl-d-glucuronic acid (Panel c) Are Also Depicted^a^

a For all systems, the ring-opening involves the C1–O bond scission leading to an acyclic product, the ring contraction transforms the pyran ring into a five-membered ring (FF or FFL), and anhydroxylopyranose (AXP or AXPL) forms via elimination. This figure has been reproduced from ref 106. Available under a CC-BY 3.0 license. Copyright 2024 L. Ayarde-Henríquez, J. Lupi, S. Dooley.

Table 1 shows the relative activation enthalpy, Δ*H* ^‡^, of xylose, 2-*O*-acetyl-xylose, and 4-*O*-methyl-d-glucuronic acid at both 0 and 298 K for ring opening/contraction and elimination across five levels of theory: M06–2X/6–311++G­(d,p) (hereafter termed DFT), CBS-QB3, G4, DLPNO–CCSD­(T)-F12/cc-pVTZ-F12//M06–2X/6–311++G­(d,p) (hereafter referred to as DLPNO), and DLPNO–CCSD­(T) with the complete basis set (CBS) extrapolation scheme (hereafter called DLPNO/CBS). The CBS extrapolation scheme strongly tends to favor the reaction progression by lowering the computed Δ*H* ^‡^ at DLPNO in 0.63 kcal mol^–1^. This MAE provides an estimation of the basis-set truncation error for the rest of the systems since the size of highly functionalized xyloses and dimers makes the DLPNO/CBS approach unaffordable, meaning that their reference level is DLPNO. Notably, the (cheaper) explicitly correlated F12 method yields energies comparable to the DLPNO/CBS reference level, contrary to benchmarks on smaller systems (up to five atoms).^131^

**Table 1:** Activation Enthalpies, Δ*H* ^‡^, in kcal mol^–1^ at 0 and 298 K for Ring-Opening, Ring Contraction, and Elimination Reactions Observed in Xylose, 2-*O*-Acetyl-xylose, and 4-*O*-Methyl-d-gluguronic Acid at Different Levels of Theory

	***T* = 0 K**					***T* = 298 K**				
**elementary reaction**	^b^Δ*H*_DFT_^‡^	^c^Δ*H*_CBS_^‡^	Δ*H*_G4_^‡^	^d^Δ*H*_DLPNO_^‡^	^e^Δ*H*_DLPNO/CBS_^‡^	^b^Δ*H*_DFT_^‡^	^c^Δ*H*_CBS_^‡^	Δ*H*_G4_^‡^	^d^Δ*H*_DLPNO_^‡^	^e^Δ*H*_DLPNO/CBS_^‡^
xylose→D‐xylose	49.68	38.19	45.55	49.36	48.86	45.54	37.30	45.54	45.35	44.85
$\mathrm{xylose}\to \mathrm{FF}1+H_{2}O$	76.21	71.20^a^	71.41	74.96	74.52	72.84	71.30^a^	71.47	71.70	71.26
$\mathrm{xylose}\to \mathrm{FF}2+H_{2}O$	70.25	56.68	65.07	68.71	68.31	70.25	56.68	65.09	65.86	65.47
$\mathrm{xylose}\to \mathrm{AXP}1+H_{2}O$	77.19	64.27	69.11	74.72	75.07	71.81	63.48	69.65	69.90	70.25
$\mathrm{xylose}\to \mathrm{AXP}2+H_{2}O$	81.68	67.01	80.73	87.47	86.99	76.07	66.46	81.08	82.43	81.95
$\mathrm{xylose}\to \mathrm{AXP}3+H_{2}O$	80.08	66.41	73.49	79.48	78.69	74.78	65.96	73.76	74.47	73.68
$\mathrm{xylose}\to \mathrm{AXP}4+H_{2}O$	81.45	67.78	74.86	81.22	80.44	76.08	67.40	75.20	76.04	75.26
$\mathrm{xylose}\to \mathrm{AXP}5+H_{2}O$	79.73	66.27	74.05	80.37	79.60	74.55	65.69	74.39	75.25	74.48
$\mathrm{xylose}\to \mathrm{AXP}6+H_{2}O$	87.20	73.70	74.38	80.87	80.63	82.00	73.27	74.85	75.44	75.20
$\mathrm{xylose}\to \mathrm{AXP}7+H_{2}O$	75.55	62.03	71.49	77.20	76.16	70.57	61.64	71.56	71.98	70.94
2‐O‐acetyl‐xylose→OADXP	45.56	43.99	43.91	45.23	44.70	42.65	43.89	43.79	41.42	40.88
$2‐O‐\mathrm{acetyl}‐\mathrm{xylose}\to \mathrm{FFL}1+CH_{3}\mathrm{COOH}$	-	-	-	-	-	-	-	-	-	-
$2‐O‐\mathrm{acetyl}‐\mathrm{xylose}\to \mathrm{FFL}2+CH_{3}\mathrm{COOH}$	71.64^a^	65.49	65.27	69.02^a^	68.71^a^	68.44^a^	65.61	65.35	64.51^a^	64.04^a^
$2‐O‐\mathrm{acetyl}‐\mathrm{xylose}\to \mathrm{AXPL}1+H_{2}O$	75.25	68.04	67.72	71.45	71.07	69.60	70.49	67.94	66.95	66.58
$2‐O‐\mathrm{acetyl}‐\mathrm{xylose}\to \mathrm{AXPL}2+CH_{3}\mathrm{COOH}$	76.33	73.76	73.28	77.68	78.15	71.17	73.58	73.80	72.87	73.35
$2‐O‐\mathrm{acetyl}‐\mathrm{xylose}\to \mathrm{AXPL}3+CH_{3}\mathrm{COOH}$	77.76	65.25	65.14	69.32	69.04	72.57	71.63	65.43	64.31	64.03
$2‐O‐\mathrm{acetyl}‐\mathrm{xylose}\to \mathrm{AXPL}4+H_{2}O$	75.77	72.43	71.94	76.50	75.72	70.84	72.72	72.19	71.72	70.94
$2‐O‐\mathrm{acetyl}‐\mathrm{xylose}\to \mathrm{AXPL}5+H_{2}O$	70.94	71.49	71.32	75.43	74.47	65.77	65.55	71.43	70.72	69.77
$2‐O‐\mathrm{acetyl}‐\mathrm{xylose}\to \mathrm{AXPL}6+H_{2}O$	79.37	73.10	72.38	77.67	76.93	74.39	74.32	72.79	72.46	71.72
$2‐O‐\mathrm{acetyl}‐\mathrm{xylose}\to \mathrm{AXPL}7+H_{2}O$	75.25	70.38	69.78	74.72	74.02	66.07	68.33	69.87	69.24	68.53
4‐O‐methyl‐D‐glucuronicacid→DGLA	51.87	47.77	47.66	51.57	51.13	47.36	47.76	47.47	47.19	46.75
$4‐O‐\mathrm{methyl}‐D‐\mathrm{glucuronic}\;\mathrm{acid}\to \mathrm{FFL}1+H_{2}O$	82.85	76.77^a^	76.70^a^	81.22	80.78	79.21	77.02^a^	76.99^a^	77.70	77.26
$4‐O‐\mathrm{methyl}‐D‐\mathrm{glucuronic}\;\mathrm{acid}\to \mathrm{FFL}2+H_{2}O$	67.35	60.91	61.01	64.29	64.71	64.66	60.94	61.03	61.69	62.11
$4‐O‐\mathrm{methyl}‐D‐\mathrm{glucuronic}\;\mathrm{acid}\to \mathrm{AXPL}1+H_{2}O$	75.64	70.19	68.79	76.13	76.40	62.58	64.08	69.27	71.14	71.41
$4‐O‐\mathrm{methyl}‐D‐\mathrm{glucuronic}\;\mathrm{acid}\to \mathrm{AXPL}2+H_{2}O$	85.70	75.67	75.29	86.12	85.60	74.73	72.85	75.10	81.92	81.40
$4‐O‐\mathrm{methyl}‐D‐\mathrm{glucuronic}\;\mathrm{aci}d\to \mathrm{AXPL}3+H_{2}O$	80.77	74.82	74.29	80.49	79.61	73.68	74.23	74.60	75.50	74.61
$4‐O‐\mathrm{methyl}‐D‐\mathrm{glucuronic}\;\mathrm{acid}\to \mathrm{AXPL}4+H_{2}O$	73.24	69.40	68.89	74.43	73.22	68.71	69.38	68.78	70.03	68.82
$4‐O‐\mathrm{methyl}‐D‐\mathrm{glucuronic}\;\mathrm{acid}\to \mathrm{AXPL}5+H_{2}O$	78.65	73.87	73.51	79.42	78.84	75.62	75.19	73.84	74.60	74.02
$4‐O‐\mathrm{methyl}‐D‐\mathrm{glucuronic}\;\mathrm{acid}\to \mathrm{AXPL}6+CH_{3}\mathrm{OH}$	79.67	72.23	72.28	79.89	79.19	81.37	75.53	72.92	75.10	74.41
$4‐O‐\mathrm{methyl}‐D‐\mathrm{glucuronic}\;\mathrm{acid}\to \mathrm{AXPL}7+CH_{3}\mathrm{OH}$	66.35	63.95	63.53	67.66	67.08	70.49	70.62	63.70	64.00	63.42

a Indicates relaxed convergence criteria.

b DFT stands for M06–2X/6–311++G­(d,p).

c CBS stands for CBS-QB3.

d DLPNO stands for DLPNO–CCSD­(T)-F12/cc-pVTZ-F12//M06–2X/6–311++G­(d,p).

e DLPNO/CBS stands for DLPNO with extrapolation to the complete basis set using cc-pVDZ and cc-pVTZ.

While the discussion of the barriers is focused on the highest temperature because the standard activation enthalpy is more appealing to pyrolysis, similar trends are observed for SCF energies. The benchmarking of the twenty-eight species’ barriers mirrors that DFT performs surprisingly well relative to methods specifically tailored for accurate thermochemistry. The DLPNO–DFT comparison unveils no significant differences (i.e., >±5.0 kcal mol^–1^), although DFT tends to slightly underestimate Δ*H* ^‡^, whereas for DFT-G4 and −CBS-QB3, 6% of the data set exceeded this threshold. Conversely, 2 and 4% of the barriers deviate significantly for DLPNO-G4 and −CBS-QB3, respectively. These findings highlight the adequacy of DFT and robustness of G4 over CBS-QB3 for informing thermodynamic quantities in pyrolysis, which is consistent with recent studies.^106^ For composite model chemistry methods, this is attributed to fundamental differences in the accuracy of CCSD­(T) and QCSD­(T) to describe correlation effects. Refer to the SI for a detailed report on barriers and associated benchmarking.

A note is deserved for TSs leading to the pyran-to-furan conversion. TS-FFL1 failed to converge for 2-*O*-acetyl-xylose at the DFT level regardless of leveraging Gaussian 16 algorithms such as synchronous transit-guided quasi-Newton (STQN) and scan. Attempts to explore the PESs using the nudged elastic band (NEB) and NEB-TS methods implemented in ORCA 5.0 proved unsuccessful in giving a good guess for this TS. Conversely, TS-FFL2 was located upon relaxing Gaussian 16 convergence criteria, i.e., *ConvF* = *M* × 10^–6^, where *M* = 3.0 × 10^5^. This approach was also adopted for xylose (at CBS-QB3) and 4-*O*-methyl-d-glucuronic acid (for both CBS-QB3 and G4 levels), using *M* = 1.2 × 10^4^ and *M* = 3.0 × 10^5^, respectively. These values are signaled by a superscript and are excluded from our analysis; see Table 1.

The analysis of the time required to compute all the reactivity indices is essential to demonstrate their computational cost-effectiveness. The wall-clock time required to calculate the electron population of C–O bonds in mono- and disaccharides shows a strong linear scaling with the size of the system (*R* ^2^ = 0.98). For glucuronoxylan, the bulkiest hemicellulose motif (180e), the wall time is approximately 14 min (mins), whereas less than 3 min were needed for the smallest structure featuring 80e (xylose). See the SI for further details. Notably, the time consumed by the other density-based parameters remained below a 0.5 min threshold for all examined species. The three descriptors were computed on a single Intel Core i7–1255U of a DELL laptop with no graphics processing unit (GPU). This underscores that the EPL models are computationally nonintensive, as deriving these polynomials requires minimal resources.

Four EPL relations are elucidated to investigate their performance across the levels of theory herein utilized. All models show a strong multilinear correlation, as evidenced by *R*_adj_^2^ = 0.80. MAE and RMSE range from 3.24 to 3.63 and 4.11–4.68 kcal mol^–1^, respectively. Surprisingly, the best-fitting polynomial corresponds to the DFT level, contrasting with CBS-QB3′s function; see Figure 2. Therefore, the DFT model is recommended as the standard tool for predicting activation enthalpies in hemicellulose depolymerization. Additionally, the G4 parametric equation presents a promising alternative, as its fitting parameters closely align with those of the DFT.

**Figure 2:**
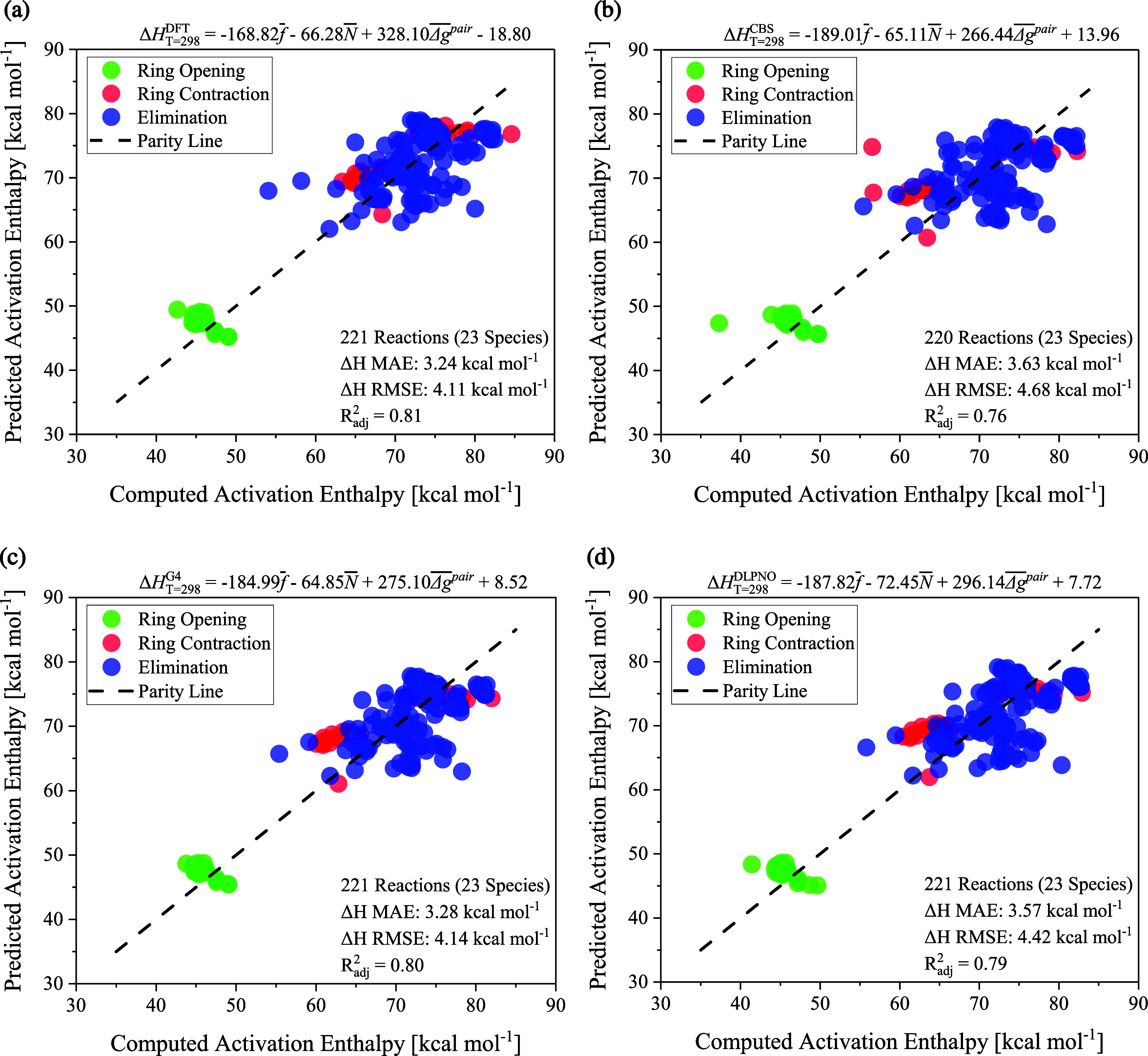
Parity plots of the Δ*H*_*T*=298_^‡^ MAE at M06–2X/6–311++G­(d,p) (Panel a), CBS-QB3 (Panel b), G4 (Panel c), and DLPNO–CCSD­(T)-F12/cc-pVTZ-F12//M06–2X/6–311++G­(d,p) (Panel d). The reactivity indices were computed from wave functions at the M06–2X/6–311++G­(d,p) level.

It is noteworthy that EPL relations accurately capture the fundamental physics governing chemical reactions, as demonstrated by the relatively high negative (169–189) and positive (266–328) coefficients of *f̅* and $\overset{®}{\Delta g^{\mathrm{pair}}\;}$ respectively. This is a promising feature aligning with chemists’ expectations since molecular sites with high values of Fukui’s functions correlate with high chemical reactivity, facilitating the identification of favorable pathways, i.e., chemical transformations with lower barriers. Similarly, the higher the bond strength index at a bonding region, the more energy is required for the system to undergo a reactive process that involves the sharing domain. Note that *N̅* behaves slightly like Fukui’s functions in the $\left\{\mathrm{f̅},\overset{®}{\Delta g^{\mathrm{pair}}\;}\right\}$ phase-space. Conversely, attempts to derive models for each reaction class yielded poor-fitting parameters, evidencing the beneficial influential nature of the computed indices when put together in a unified set.

Although the predictive accuracy of these models is far from ideal, sophisticated machine learning (ML) algorithms, such as Artificial Neural Networks (ANN) and Gaussian Process Regression (GPR), tailored to predict thermochemical properties, show comparable performance. This highlights the inherent complexities of studying the activated complex, particularly in informing the thermochemical features of TSs. For instance, Singh et al.^160^ built an ML-based tool for estimating gas-phase barriers of dehydrogenation and diatomic molecule dissociations on a metal surface, reporting MAE about 5 kcal mol^–1^. Recently, Stuyver and Coley^161^ trained ANN for activation enthalpy prediction of both bimolecular nucleophilic substitution (S_N_2) and elimination (E2) reactions in vacuum. Regardless of the apparent simplicity of the systems investigated, they found MAE and RMSE of ≈3 and ≈4 kcal mol^–1^, respectively. Similarly, Green and collaborators^162^ developed an ANN-based model for predicting barriers of intramolecular rearrangement, ring-opening, and hydrogen transfer reactions in closed-shell organic molecules in the gas phase, observing MAE and RMSE of approximately 3 and 5 kcal mol^–1^, respectively. See Table 2. These insights suggest that EPL relations can serve as a reliable framework for probing the hemicellulose motifs’ PES, thereby accelerating the derivation of reaction networks by rapidly disregarding energetically unfeasible elementary steps.

**Table 2:** Performance of EPL and ML-Driven Models

study	ML technique	molecular system (gas phase)	reaction class	data points	MAE [kcal mol^‑1^]	parameters
Singh et al.	ANN	NH_3_, CH_4_, H_2_O	dehydrogenation	315	5.07	9
		N_2_, O_2_	dissociation			
Stuyver and Coley	ANN	R_3_C-CX_3_ + Y^®^ (R = H, CN, NO_2_, NH_2_, CH_3_; X = F, Cl, Br; Y = H, X)	S_N_2	791	2.65	6
		R_2_C-CR_2_ + X^®^	E2			
Green et al.	ANN	C/H/O/Metal	rearrangement	<17000	2.57	5
			ring-opening			
			H transfer			
this work	-	C/H/O	ring-opening	221	3.24	3
			ring contraction			
			H transfer			

For all EPL relations, the MAE arithmetic mean distributes smoothly across the levels of theory, with values approximately ranging from 7.5 to 9.4 and 2.5–3.7 kcal mol^–1^ for 10 and 90% of the data points, respectively, whereas the standard deviation (SD) varies from 0.2 to 0.5 kcal mol^–1^ for the 95%. The marginal high values of SD are attributed to the sample low population representing 5% of the data set. In such cases, SD was estimated using Hozo’s methodology.^163^ See Figure 3.

**Figure 3:**
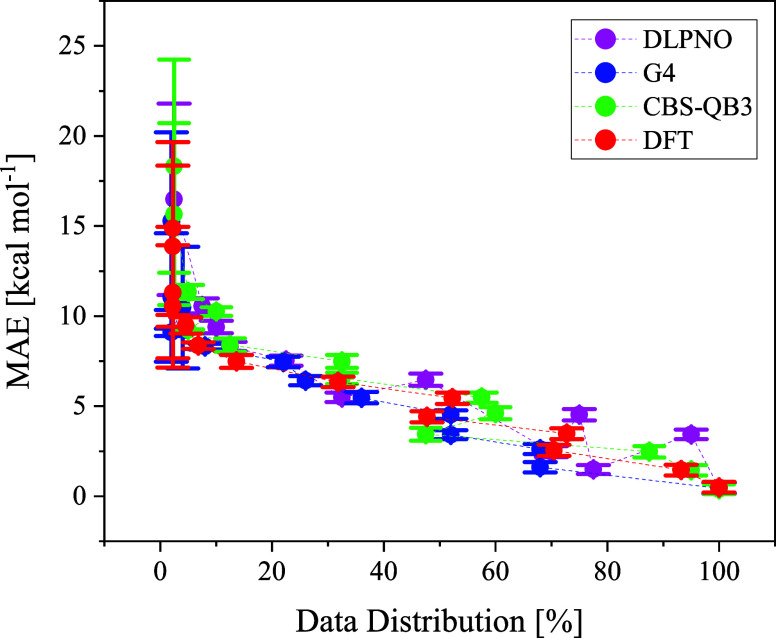
Distribution of MAE’s mean and standard deviation across the entire data for each Evans–Polanyi-like model. DFT stands for M06–2X/6–311++G­(d,p) and DLPNO for DLPNO–CCSD­(T)-F12/cc-pVTZ-F12//M06–2X/6–311++G­(d,p).

Five naturally occurring saccharides, including two monomers, β-d-glucopyranose (glucose) and 5-carboxy-xylose, and three dimers, 1,4-β-xylobiose (xylobiose), β-d-xylopyranose,1,4-β-glucopyranose (xyloglucan), and 4-methoxy-5-carboxy-β-d-xylopyranose,1,4-β-d-xylopyranose (glucuronoxylan), totalizing 50 elementary reactions, served as the validating set. Although no dimer was used in the EPL derivation stage, the performance of the derived models is promising. The MAE and RMSE of predicted barriers fall within 3.2–3.5 and 3.9–4.4 kcal mol^–1^, respectively. Notably, the highest MAE corresponds to DLPNO, whereas the lowest is associated with DFT, as depicted in Figure 4.

**Figure 4:**
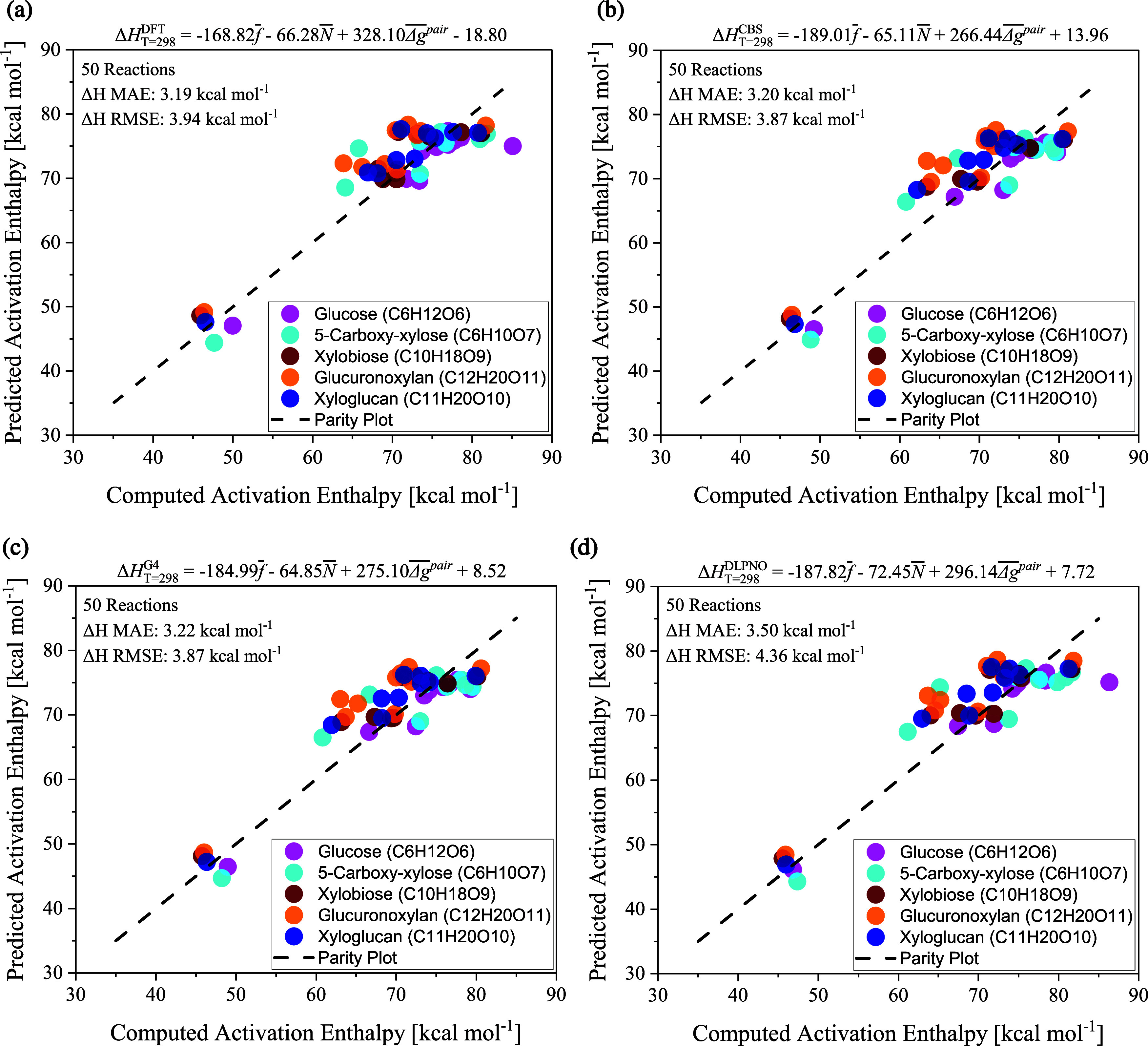
Predicted standard activation enthalpies of the validating set, including five real-world saccharides: glucose, 5-carboxy-xylose, xylobiose, xyloglucan, and glucuronoxylan, showing the performance of DFT (Panel a), CBS (Panel b), G4 (Panel c), and DLPNO (Panel d). DFT stands for M06–2X/6–311++G­(d,p) and DLPNO for DLPNO–CCSD­(T)-F12/cc-pVTZ-F12//M06–2X/6–311++G­(d,p).

### 3.2 Sensitivity Analysis

EPL polynomials map computed standard activation enthalpies to their predicted values. Thus, assessing how the system size and the reaction type influence the accuracy of computed enthalpies is crucial, as the effects of these variables can propagate to the EPL models and impact their predictive capabilities. A sensitivity analysis of the levels of theory accuracy to inform barriers can provide insights into the EPL polynomials’ performance. The size of the studied systems (*N*) has a negligible influence on the computed barriers across all levels of theory for *N* > 80e. This means that significant discrepancies in the standard activation enthalpies between CBS-QB3 and other methods are observed exclusively for reactions involving xylose, as depicted in Figure 5. Conversely, consistent results are found for the ring-opening and elimination reaction classes, see Figure 5 Panel a and Panels c and d, respectively. In contrast, the ring contraction barriers at DFT differ from those obtained from other levels by approximately 5.0 kcal mol^–1^, which is herein considered the upper limit for significant differences; see Figure 5, Panel b. This suggests that special care should be undertaken when describing this reaction class with, at least, the DFT method due to ring contraction’s complexities. Furthermore, the functional groups have a null impact on the performance of the methods (see Figure 5, Panel d). These outcomes are consistent with the EPL functions’ features, providing a plausible rationale for the relatively small dispersion in the predicted enthalpies of ring-opening (see Figures 2 and 4).

**Figure 5:**
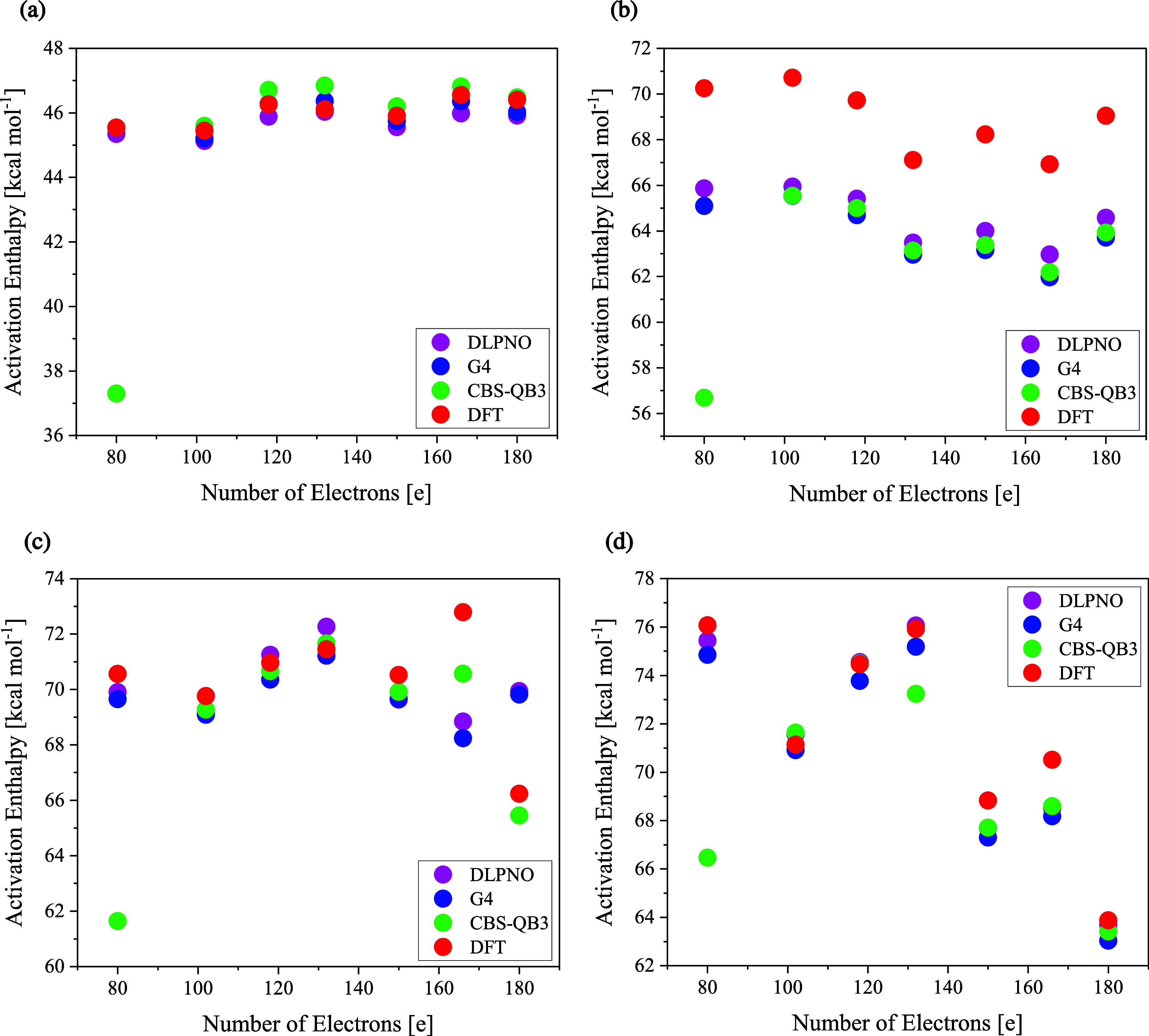
Δ*H*_*T*=298_^‡^ as a function of *N* for ring-opening (Panel a), ring contraction (Panel b), and elimination (Panels c and d). The barriers correspond to xylose (*N* = 80e), 3-*O*-acetyl-xylose (*N* = 102e), 3-*O*-acetyl-5-methoxy-xylose (*N* = 118e), 3-O-acetyl-5-*O*-acetyl (*N* = 132e), xylobiose (*N* = 150e), xyloglucan (*N* = 166e), and glucuronoxylan (*N* = 180e). DFT stands for M06–2X/6–311++G­(d,p) and DLPNO for DLPNO–CCSD­(T)-F12/cc-pVTZ-F12//M06–2X/6–311++G­(d,p). The processes shown in Panels a–c involve no functional group; in contrast, Panel d depicts the elimination reactions for which the substituent is active in the case of the dimers: xylobiose, xyloglucan, and glucuronoxylan. For xylose, CBS-QB3 significantly underestimates the barriers of the three reaction classes relative to DFT, G4, and DLPNO. For *N* > 80e, the highest disparities (≈5.0 kcal mol^–1^) in the computed barriers correspond to ring contraction observed between DFT and the other methods.

Conversely, xylose exhibits high structural flexibility and numerous hydroxyl groups, allowing it to exist in a variety of (metastable) conformers. This means that the initial conformer choice is key, as selecting a low-populated structure can lead to inaccuracies in computed thermochemical properties and, consequently, influence the performance of EPL models. Recently, Ballotta and colleagues^164^ have thoroughly explored the conformational space of xylose using metadynamics and quantum chemistry methods in the 298–1068 K range. Based on their findings, we selected the most stable and populated conformer across the pyrolytic temperatures in this work.

### 3.3 Benchmark

Figure 6 shows six linear models for extrapolating the computed standard activation enthalpy from lower to higher levels of theory. These relations comprise the full set of studied systems, i.e., 28 species. For all cases, the linearity is markedly strong, as demonstrated by *R* ^2^ > 0.95. This is also evidenced by the slope of each polynomial, which is very close to +1.0. Extrapolating from DFT to both G4 and DLPNO entails a relatively low MAE of 1.13 kcal mol^–1^ in both situations, contrasting with CBS-QB3, for which MAE = 1.63 kcal mol^–1^ is observed. Conversely, MAE of about 1.0 kcal mol^–1^ should be expected when estimating barriers at DLPNO and G4 from values calculated at CBS-QB3. The barriers computed at G4 and DLPNO show the strongest linear correlation, with MAE of 0.61 kcal mol^–1^. A fair agreement between barriers at CBS-QB3, G4, and DLPNO is expected since these methods have been developed for accurate thermochemistry. However, the performance of DFT across 270 chemical reactions, including three different classes, is notable and underscores this method’s suitability for investigating hemicellulose pyrolysis. In this context, analogous expressions with *R* ^2^ ranging from 0.93 to 0.97 have been derived using cheaper DFT levels, specifically, B3LYP and ωB97X-D in combination with the 6–31+G­(d,p) basis set, to accelerate the exploration of PESs and facilitate the initial stages of kinetic model development by disregarding processes with too high energy barriers. See the SI for further details.

**Figure 6:**
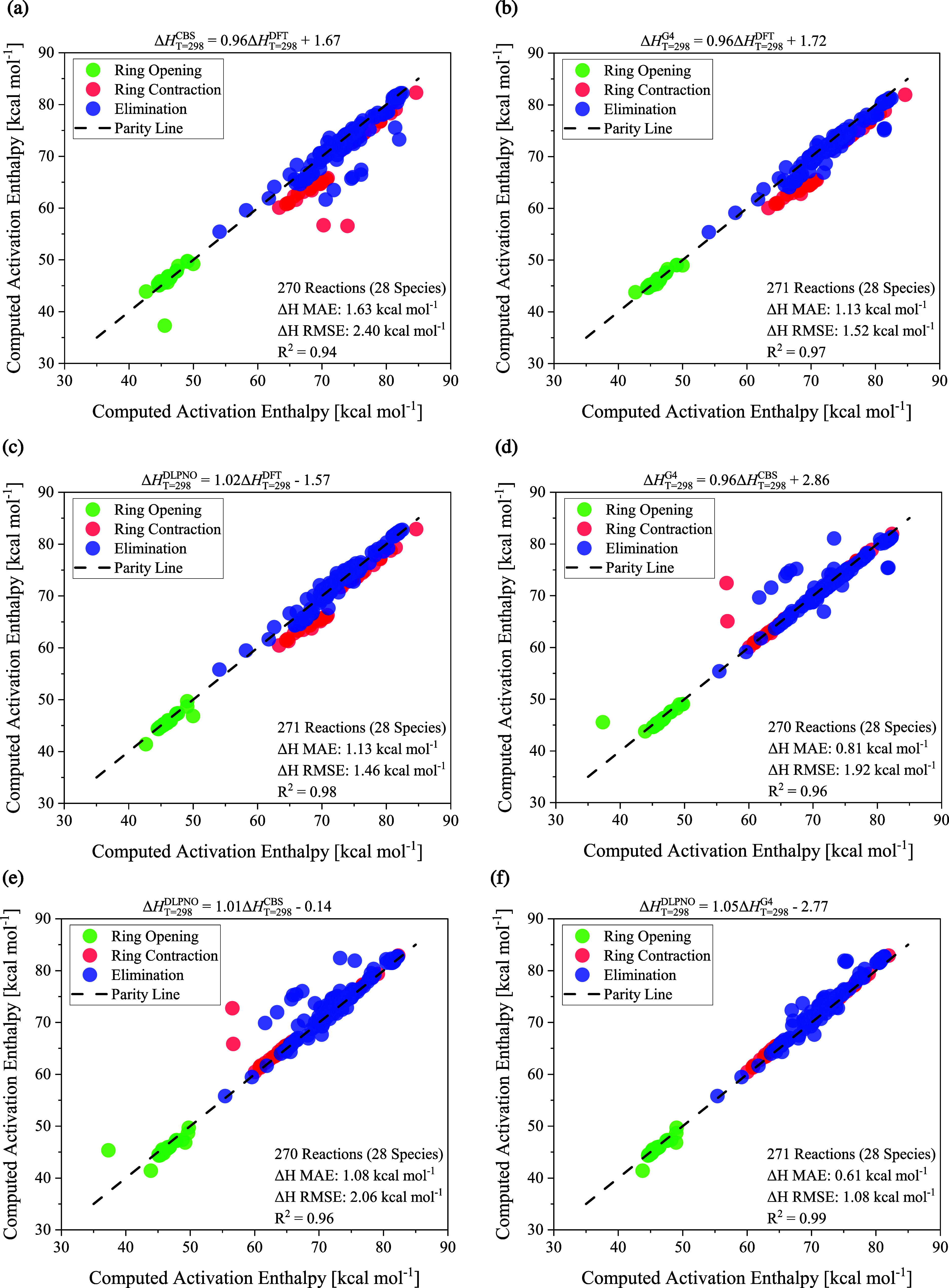
Parity plots of the Δ*H*_*T*=298_^‡^ MAE for extrapolating computed barriers from M06–2X/6–311++G­(d,p) to CBS-QB3 (Panel a), M06–2X/6–311++G­(d,p) to G4 (Panel b), M06–2X/6–311++G­(d,p) to DLPNO–CCSD­(T)-F12/cc-pVTZ-F12//M06–2X/6–311++G­(d,p) (Panel c), CBS-QB3 to G4 (Panel d), CBS-QB3 to DLPNO–CCSD­(T)-F12/cc-pVTZ-F12//M06–2X/6–311++G­(d,p) (Panel e), and G4 to DLPNO–CCSD­(T)-F12/cc-pVTZ-F12//M06–2X/6–311++G­(d,p) (Panel f).

## 4 Conclusions

We have derived Evans–Polanyi-like (EPL) relations to rapidly inform the standard activation enthalpy of three ubiquitous reaction classes established as fundamental to understanding hemicellulose pyrolysis through chemical kinetic models: ring-opening, ring contraction, and elimination. EPL models carefully replaced the degrees of freedom of product(s) by computationally cheap local and global electron-density-based chemical descriptors, including Fukui’s functions (*f*), electron population of C–O bonds (*N*), and the gross intrinsic strength bond index (Δ*g* ^pair^), characterizing reactant’s reactivity. These parameters have demonstrated high stability, showing a negligible dependence on both the level of theory and the quality of the numerical integration grid. A set of 270 reactions observed in twenty-eight functionalized β-d-xylopyranoses, the hemicellulose building block, was used under the 20–80% partition scheme in the validation-derivation process. Four EPL polynomials were elucidated using regression analysis for barrier prediction at M06–2X/6–311++G­(d,p), CBS-QB3, G4, and DLPNO–CCSD­(T)-F12/cc-pVTZ-F12//M06–2X/6–311++G­(d,p): Δ*H* ^DFT^ = – 168.82*f̅* – 66.28*N̅* + 328.10Δ̅*g* ^pair^ – 18.80, Δ*H* ^CBS^ = – 189.01*f̅*– 65.11*N̅* + 266.44Δ̅*g* ^pair^ + 13.96, Δ*H* ^G4^ = – 184.99*f̅* – 64.85*N̅* + 275.10Δ̅*g* ^pair^ + 8.52, and Δ*H* ^DLPNO^ = – 187.82*f̅* – 72.45*N̅* + 296.14Δ̅*g* ^pair^ + 7.72, respectively. The best-fitting models, at DFT and G4, yielded MAE and RMSE of about 3.3 and 4.1 kcal mol^–1^, respectively, contrasting with CBS-QB3′s function, which exhibited the highest MAE = 3.6 and RMSE = 4.7 kcal mol^–1^ values. Notably, an adjusted coefficient of determination around 0.80 was observed for all EPL models, underscoring their reasonable predictive capabilities. Regardless of their simplicity, such relations captured the underlying fundamental physics of reactive processes, as demonstrated by the highly negative and positive coefficients of Fukui’s functions and intrinsic strength bond index, respectively. Extrapolation expressions were derived upon benchmarking the computed standard activation enthalpies, enabling the estimation of this quantity at higher levels from lower ones: Δ*H* ^CBS^ = 0.96Δ*H* ^DFT^ + 1.67, Δ*H* ^G4^ = 0.96Δ*H* ^DFT^ + 1.72, Δ*H* ^DLPNO^ = 1.02Δ*H* ^DFT^ – 1.57, Δ*H* ^G4^ = 0.96Δ*H* ^CBS^ + 2.86, Δ*H* ^DLPNO^ = 1.01Δ*H* ^CBS^ – 0.14, and Δ*H* ^DLPNO^ = 1.05Δ*H* ^G4^ – 2.77. These linear models exhibited a coefficient of determination that fell within 0.94–0.98. In addition, analogous polynomials with comparable predictive accuracy were elucidated for lower-cost DFT levels to further accelerate the early developing stages of kinetic models. These models are reported in the Supporting Information (SI). The extrapolation of DPLNO barriers to the complete basis set limit strongly tended to lower them by 0.63 kcal mol^–1^, indicating that the cheaper explicit correlated method F12 provided accurate energies. EPL formulations were developed to explore the potential energy surface of hemicellulose pyrolysis effortlessly, aiming to aid in developing detailed chemical kinetic models. This is a crucial step toward the faster commercialization of bioproducts at large scales.

## Supplementary Material





## Notes

Optimized Cartesian coordinates for reactants and transition states of both monomers and dimers involved in ring-opening, ring contraction, and elimination reaction classes at the M06–2X/6–311++G­(d,p) DFT level (xyz); $T_{1}$ diagnostic values for reactants and transition states; benchmark of the activation enthalpy at 0 and 298 K computed at four levels, including M06–2X/6–311++G­(d,p), CBS-QB3, G4, and DLPNO–CCSD­(T)-F12/cc-pVTZ-F12//M06–2X/6–311++G­(d,p), linear model for barriers estimation at CBS-QB3, G4, and DLPNO–CCSD­(T)-F12/cc-pVTZ-F12//M06–2X/6–311++G­(d,p) from ωB97X-D and B3LYP in combination with the 6–31+G­(d,p) split-valence basis set (PDF)
Optimized coordinates *XYZ* (ZIP)
