# Supplementary material for: Evans–Polanyi-like Formulations for Rapidly Predicting Key Depolymerization Barriers in Xylopyranoses: Toward the Faster Development of Kinetic Models for Hemicellulose Pyrolysis
Source: J Phys Chem A. 2025 May 14;129(21):4767–85. doi: 10.1021/acs.jpca.5c00675 (PMC12128103; doi:10.1021/acs.jpca.5c00675)
Supplement: Supplementary file 1 [file jp5c00675_si_001.pdf]

Supporting Information

Evans–Polanyi-like Formulations for Rapidly Predicting Key Depolymerization Barriers in Xylopyranoses: Toward the Faster Development of Kinetic Models for Hemicellulose Pyrolysis

Leandro Ayarde-Henríquez,*[ab] Jacopo Lupi,[c] Bernardo Ballotta,[ab] and Stephen Dooley[ab]

[a]School of Physics, Trinity College Dublin, Dublin 2 D02 PN40, Ireland.
[b]AMBER, Advanced Materials and BioEngineering Research Centre, Dublin 2 D02 PN40, Ireland.
[c]CNR-ICCOM, Consiglio Nazionale delle Ricerche, via Giuseppe Moruzzi 1, Pisa I-56124, Italy.

Correspondence to: leandro.ayarde@tcd.ie

Contents

1. Table S1. Thermochemistry at T = 298 K and P =1 atm initial reactions at the M06-2X/6-311++G(d,p) DFT level. Electronic energies, ε, and corrected enthalpies, H_{corr}, in Hartree. S1
2. Table S2. $\mathcal{T}1$ diagnostic....................S3
3. Table S3. Activation enthalpies, $\Delta H^{\ddagger}$, in kcal mol^{-1} at 0 and 298 K for ring-opening, ring contraction and elimination at different levels of theory. S6
4. Table S4. Activation enthalpies, $\Delta H^{\ddagger}$, in kcal mol^{-1} at 0 and 298 K for ring-opening, ring contraction and elimination at different levels of theory. S7
5. Table S5. Activation enthalpies, $\Delta H^{\ddagger}$, in kcal mol^{-1} at 0 and 298 K for ring-opening, ring contraction and elimination at different levels of theory. S8
6. Table S6. Activation enthalpies, $\Delta H^{\ddagger}$, in kcal mol^{-1} at 0 and 298 K for ring-opening, ring contraction and elimination at different levels of theory. S9
7. Table S7. Activation enthalpies, $\Delta H^{\ddagger}$, in kcal mol^{-1} at 0 and 298 K for ring-opening, ring contraction and elimination at different levels of theory. ...S10
8. Table S8. Activation enthalpies, $\Delta H^{\ddagger}$, in kcal mol^{-1} at 0 and 298 K for ring-opening, ring contraction and elimination at different levels of theory. ...S11
9. Table S9. Activation enthalpies, $\Delta H^{\ddagger}$, in kcal mol^{-1} at 0 and 298 K for ring-opening, ring contraction and elimination at different levels of theory. ...S12
10. Table S10. Activation enthalpies, $\Delta H^{\ddagger}$, in kcal mol^{-1} at 0 and 298 K for ring-opening, ring contraction and elimination at different levels of theory. S13
11. Table S11. Activation enthalpies, $\Delta H^{\ddagger}$, in kcal mol^{-1} at 0 and 298 K for ring-opening, ring contraction and elimination at different levels of theory. S14
12. Extrapolation relations for extrapolating standard activation enthalpies from B3LYP/6-31+G(d,p) to CBS-QB3, G4, and DLPNO-CCSD(T)-F12/cc-pVTZ-F12//M06-2X/6-311++G(d,p).....................S14
13. Extrapolation relations for extrapolating standard activation enthalpies from ωB97X-D/6-31+G(d,p) to CBS-QB3, G4, and DLPNO-CCSD(T)-F12/cc-pVTZ-F12//M06-2X/6-311++G(d,p).....................S14
14. Wall-clock time required to calculate the electron population of C-O bonds across mono- and di-saccharides species.....................S15
15. Benchmark.....................S15

1. Table S1. Thermochemistry at T = 298 K and P = 1.0 atm at the M06-2X/6-311++G(d,p) DFT level. Electronic energies, ε, and corrected enthalpies, $\varepsilon+H_{corr}$, in Hartree.

Species	ε	$\varepsilon+H_{corr}$	Species	ε	$\varepsilon+H_{corr}$	Species	ε	$\varepsilon+H_{corr}$
Xylose	-572.6117308	-572.434460	2-O-acetyl-xylose	-725.2484786	-725.029683	4-O-methyl-D-glucuronic acid	-800.467725	-800.242714
TS-DXP	-572.5325529	-572.361883	TS-OADXP	-725.174280	-724.961737	TS-DGLA	-800.3850586	-800.167237
TS-FF1	-572.4902875	-572.318389	TS-FFL1	-	-	TS-FFL1	-800.3356992	-800.116487
TS-FF2	-572.4950926	-572.322510	TS-FFL2	-	-	TS-FFL2	-800.3604035	-800.139677
TS-AXP1	-572.4887132	-572.320029	TS-AXPL1	-725.1285585	-724.918774	TS-AXPL1	-800.3619864	-800.142984
TS-AXP2	-572.4815621	-572.313238	TS-AXPL2	-725.1246625	-724.914439	TS-AXPL2	-800.3407572	-800.123625
TS-AXP3	-572.4841139	-572.315283	TS-AXPL3	-725.1295851	-724.918532	TS-AXPL3	-800.3423832	-800.125290
TS-AXP4	-572.4819382	-572.313207	TS-AXPL4	-725.1277349	-724.916794	TS-AXPL4	-800.3510086	-800.133214
TS-AXP5	-572.4846759	-572.315662	TS-AXPL5	-725.1354284	-724.924870	TS-AXPL5	-800.3390057	-800.122211
TS-AXP6	-572.4727651	-572.303789	TS-AXPL6	-725.1219994	-724.911131	TS-AXPL6	-800.331154	-800.113047
TS-AXP7	-572.4913277	-572.322007	TS-AXPL7	-725.135793	-724.924393	TS-AXPL7	-800.3471905	-800.130384
2-Methoxy-xylose	-611.8977762	-611.690862	3-Methoxy-xylose	-611.8973637	-611.690490	4-Methoxy-xylose	-611.9003657	-611.693328
TS-acyclic-product	-611.8181134	-611.617591	TS-acyclic-product	-611.8197765	-611.619266	TS-acyclic-product	-611.8215526	-611.621074
TS-FFL1	-	-	TS-FFL1	-611.7752497	-611.574088	TS-FFL1	-611.7772102	-611.575483
TS-FFL2	-	-	TS-FFL2	-611.7818217	-611.579504	TS-FFL2	-611.7840984	-611.581694
TS-AXPL1	-611.7744475	-611.576126	TS-AXPL1	-611.7770614	-611.578758	TS-AXPL1	-611.7784798	-611.579345

TS-AXPL2	-611.7757585	-611.577114	TS-AXPL2	-611.7693555	-611.571383	TS-AXPL2	-611.7711672	-611.573080
TS-AXPL3	-611.7695565	-611.571251	TS-AXPL3	-611.7747443	-611.575310	TS-AXPL3	-611.7722716	-611.573793
TS-AXPL4	-611.770166	-611.571733	TS-AXPL4	-611.7726242	-611.573572	TS-AXPL4	-611.7707713	-611.572235
TS-AXPL5	-611.77524	-611.575487	TS-AXPL5	-611.7716703	-611.572869	TS-AXPL5	-611.7730258	-611.574292
TS-AXPL6	-611.7637483	-611.563461	TS-AXPL6	-611.7582583	-611.559852	TS-AXPL6	-611.7613431	-611.562515
TS-AXPL7	-611.7789806	-611.7789806	TS-AXPL7	-611.7810174	-611.581730	TS-AXPL7	-611.7796883	-611.580631
5-Methoxy-xylose	-687.1327773	-686.919943	5-Carboxy-xylose	-761.1864021	-760.990979	Glucose	-687.1460961	-686.932577
TS-acyclic-product	-687.0542645	-686.848170	TS-acyclic-product	-761.1032535	-760.915016	TS-acyclic-product	-687.0635617	-686.856558
TS-FFL1	-	-	TS-FFL1	-761.0501737	-760.860531	TS-FFL1	-687.0172926	-686.809342
TS-FFL2	-687.0179909	-686.809577	TS-FFL2	-761.0798727	-760.888832	TS-FFL2	-687.0270813	-686.818113
TS-AXPL1	-686.9989082	-686.794601	TS-AXPL1	-761.0790176	-760.889698	TS-AXPL1	-687.0208443	-686.815077
TS-AXPL2	-686.996828	-686.793188	TS-AXPL2	-761.0508238	-760.861937	TS-AXPL2	-687.017376	-686.812208
TS-AXPL3	-687.0044209	-686.799891	TS-AXPL3	-761.0561615	-760.868763	TS-AXPL3	-687.0135137	-686.808707
TS-AXPL4	-687.0047763	-686.800093	TS-AXPL4	-761.0619564	-760.873967	TS-AXPL4	-687.0126671	-686.807324
TS-AXPL5	-687.0046591	-686.800075	TS-AXPL5	-761.057001	-760.869778	TS-AXPL5	-687.0123961	-686.807270
TS-AXPL6	-686.9948503	-686.789653	TS-AXPL6	-761.0505673	-760.861551	TS-AXPL6	-686.9996581	-686.794641
TS-AXPL7	-687.0106807	-686.806163	TS-AXPL7	-761.0610876	-760.873914	TS-AXPL7	-687.0211933	-686.815609
3-O-acetyl-xylose	-725.2452483	-725.026930	4-O-acetyl-xylose	-725.256255	-725.037659	5-O-acetyl-xylose	-800.4757334	-800.251583
TS-acyclic-product	-725.1664244	-724.954435	TS-acyclic-product	-725.1772074	-724.965137	TS-acyclic-product	-800.3973128	-800.180088
TS-FFL1	-725.1206173	-724.907835	TS-FFL1	-725.1320617	-724.918815	TS-FFL1	-800.3462922	-800.127679
TS-FFL2	-725.1279863	-724.914152	TS-FFL2	-725.1387391	-724.924782	TS-FFL2	-800.3656931	-800.145979
TS-AXPL1	-725.1238734	-724.913932	TS-AXPL1	-725.1498345	-724.939283	TS-AXPL1	-800.3479567	-800.131625
TS-AXPL2	-725.1236053	-724.913539	TS-AXPL2	-725.1347544	-724.924871	TS-AXPL2	-800.3386026	-800.123568
TS-AXPL3	-725.1318714	-724.921952	TS-AXPL3	-725.1305216	-724.920000	TS-AXPL3	-800.3486615	-800.132402
TS-AXPL4	-725.129100	-724.919140	TS-AXPL4	-725.1346504	-724.922858	TS-AXPL4	-800.3481356	-800.132017
TS-AXPL5	-725.1230311	-724.912117	TS-AXPL5	-725.1310449	-724.920313	TS-AXPL5	-800.3481238	-800.132204
TS-AXPL6	-725.1063369	-724.896463	TS-AXPL6	-725.116595	-724.906336	TS-AXPL6	-800.3397756	-800.122533
TS-AXPL7	-725.1261239	-724.915659	TS-AXPL7	-725.1388384	-724.927969	TS-AXPL7	-800.3523299	-800.136521
3-Methoxy-5-methoy-xylose	-726.4187028	-726.176081	3-Methoxy-5-carboxy-xylose	-800.4722111	-800.246996	3-Methoxy-5-O-acetyl-xylose	-839.7617049	-839.507849
TS-acyclic-product	-726.338584	-726.102634	TS-acyclic-product	-800.3868033	-800.168792	TS-acyclic-product	-839.6815874	-839.434535
TS-FFL1	-726.2921083	-726.055728	TS-FFL1	-800.3360363	-800.117219	TS-FFL1	-839.6314512	-839.383524
TS-FF2	-726.3052586	-726.066992	TS-FF2	-800.366949	-800.146040	TS-FF2	-839.6527953	-839.403204
TS-AXPL1	-726.2860702	-726.052166	TS-AXPL1	-800.3621838	-800.143442	TS-AXPL1	-839.635207	-839.389387
TS-AXPL2	-726.2849228	-726.051415	TS-AXPL2	-800.3388567	-800.120476	TS-AXPL2	-839.6339645	-839.388186
TS-AXPL3	-726.2953105	-726.060355	TS-AXPL3	-800.3476283	-800.129686	TS-AXPL3	-839.6401714	-839.393370
TS-AXPL4	-726.2956813	-726.060548	TS-AXPL4	-800.3491405	-800.131734	TS-AXPL4	-839.6395015	-839.392868
TS-AXPL5	-726.2917194	-726.057430	TS-AXPL5	-800.3443943	-800.127346	TS-AXPL5	-839.6352221	-839.389402
TS-AXPL6	-726.2806699	-726.046218	TS-AXPL6	-800.3359239	-800.117381	TS-AXPL6	-839.6255441	-839.378830
TS-AXPL7	-726.2967998	-726.062539	TS-AXPL7	-800.3472283	-800.130333	TS-AXPL7	-839.6386901	-839.393230
3-O-acetyl-5-methoxy-xylose	-839.7656715	-839.511643	3-O-acetyl-5-carboxy-xylose	-913.8180987	-913.581566	3-O-acetyl-5-O-acetyl-xylose	-953.1077809	-952.842548
TS-acyclic-product	-839.6852031	-839.438017	TS-acyclic-product	-913.7316637	-913.5022	TS-acyclic-product	-953.027312	-952.769062
TS-FFL1	-839.6375847	-839.389658	TS-FFL1	-913.6771752	-913.446707	TS-FFL1	-952.9760073	-952.716557
TS-FFL2	-839.6502934	-839.400637	TS-FFL2	-913.7105617	-913.478283	TS-FFL2	-952.9965216	-952.735614
TS-AXPL1	-839.6325441	-839.387052	TS-AXPL1	-913.6963317	-913.466394	TS-AXPL1	-952.9807507	-952.723296
TS-AXPL2	-839.6391184	-839.393083	TS-AXPL2	-913.6933191	-913.462843	TS-AXPL2	-952.9786857	-952.721571
TS-AXPL3	-839.6501186	-839.404554	TS-AXPL3	-913.7006394	-913.472318	TS-AXPL3	-952.9905375	-952.733661
TS-AXPL4	-839.6501169	-839.404532	TS-AXPL4	-913.7009442	-913.47266	TS-AXPL4	-952.9911206	-952.734255
TS-AXPL5	-839.6425146	-839.396102	TS-AXPL5	-913.6939467	-913.464896	TS-AXPL5	-952.9850153	-952.727097
TS-AXPL6	-839.627627	-839.381631	TS-AXPL6	-913.6820666	-913.45214	TS-AXPL6	-952.9715144	-952.713377
TS-AXPL7	-839.6444803	-839.398654	TS-AXPL7	-913.6943104	-913.465905	TS-AXPL7	-952.9858688	-952.728688
3Methoxy-4methoxy-5methoxy-xylose	-765.7040084	-765.431873	3Methoxy-4methoxy-5carboxy-xylose	-839.7533796	-839.498715	3O-acetyl-4methoxy-5methoxy-xylose	-879.0523918	-878.768726
TS-acyclic-product	-765.6250031	-765.359412	TS-acyclic-product	-839.6707719	-839.423216	TS-acyclic-product	-878.9731611	-878.696209
TS-FFL1	-765.5781964	-765.312147	TS-FFL1	-839.6212087	-839.372771	TS-FFL1	-878.924943	-878.647188
TS-FFL2	-765.5910837	-765.323274	TS-FFL2	-839.6465668	-839.396016	TS-FFL2	-878.9370152	-878.657607
TS-AXPL1	-765.5774361	-765.312836	TS-AXPL1	-839.6548025	-839.405973	TS-AXPL1	-878.9241968	-878.647553
TS-AXPL2	-765.5785374	-765.314566	TS-AXPL2	-839.6293923	-839.382529	TS-AXPL2	-878.930347	-878.65357
TS-AXPL3	-765.5834633	-765.318878	TS-AXPL3	-839.6341125	-839.386423	TS-AXPL3	-878.9377182	-878.662418
TS-AXPL4	-765.5810783	-765.316732	TS-AXPL4	-839.6383686	-839.390878	TS-AXPL4	-878.9368736	-878.661505
TS-AXPL5	-765.5777245	-765.313851	TS-AXPL5	-839.6262318	-839.379739	TS-AXPL5	-878.9290174	-878.653018
TS-AXPL6	-765.5654333	-765.301745	TS-AXPL6	-839.6163367	-839.368915	TS-AXPL6	-878.9137141	-878.63801
TS-AXPL7	-765.5834141	-765.319565	TS-AXPL7	-839.6336498	-839.387098	TS-AXPL7	-878.9336369	-878.65726
3O-acetyl-4methoxy-5carboxy-xylose	-953.1008181	-952.83473	4-Methoxy-5-methoxy-xylose	-726.4188687	-726.176262	4-Methoxy-5-O-acetyl-xylose	-839.7623215	-839.508432
TS-acyclic-product	-953.0180778	-952.759144	TS-acyclic-product	-726.3410391	-726.105195	TS-acyclic-product	-839.6844995	-839.437483
TS-FFL1	-952.9664121	-952.706206	TS-FFL1	-726.2939717	-726.057059	TS-FFL1	-	-
TS-FFL2	-952.991495	-952.72964	TS-FFL2	-726.3044924	-726.066284	TS-FFL2	-839.6521712	-839.402657
TS-AXPL1	-953.0088774	-952.748539	TS-AXPL1	-726.2897838	-726.054586	TS-AXPL1	-839.6399558	-839.392321
TS-AXPL2	-952.9828367	-952.723666	TS-AXPL2	-726.2866959	-726.052957	TS-AXPL2	-839.6285805	-839.383557
TS-AXPL3	-952.9845627	-952.726974	TS-AXPL3	-726.2926052	-726.058388	TS-AXPL3	-839.6368401	-839.390916
TS-AXPL4	-952.9863683	-952.728608	TS-AXPL4	-726.290059	-726.05602	TS-AXPL4	-839.6339992	-839.388432
TS-AXPL5	-952.9764474	-952.717972	TS-AXPL5	-726.291006	-726.05689	TS-AXPL5	-839.6348256	-839.389299
TS-AXPL6	-952.9640859	-952.705163	TS-AXPL6	-726.2801414	-726.045265	TS-AXPL6	-839.6257519	-839.378671
TS-AXPL7	-952.9808892	-952.723013	TS-AXPL7	-726.2975454	-726.063315	TS-AXPL7	-839.6402036	-839.394208

4-O-acetyl-5-methoxy-xylose	-839.7709902	-839.517094	Xylobiose	-1068.807655	-1068.479397	Xyloglucan	-1183.338058	-1182.973701
TS-acyclic-product	-839.6924304	-839.445233	TS-acyclic-product	-1068.727935	-1068.406237	TS-acyclic-product	-1183.256838	-1182.899517
TS-FFL1	-839.6475402	-839.399142	TS-FFL1	-1068.680857	-1068.358021	TS-FFL1	-1183.208577	-1182.850001
TS-FFL2	-839.6633865	-839.413504	TS-FFL2	-1068.694416	-1068.370661	TS-FFL2	-1183.22712	-1182.867058
TS-AXPL1	-839.6611164	-839.414253	TS-AXPL1	-1068.68341	-1068.36364	TS-AXPL1	-1183.213368	-1182.857707
TS-AXPL2	-839.6479331	-839.402434	TS-AXPL2	-1068.689568	-1068.36971	TS-AXPL2	-1183.217338	-1182.861337
TS-AXPL3	-839.6518667	-839.405244	TS-AXPL3	-1068.688831	-1068.366673	TS-AXPL3	-1183.217996	-1182.860368
TS-AXPL4	-839.6523015	-839.404924	TS-AXPL4	-1068.680582	-1068.360335	TS-AXPL4	-1183.209716	-1182.853595
TS-AXPL5	-839.6445318	-839.398711	TS-AXPL5	-1068.683038	-1068.362868	TS-AXPL5	-1183.211337	-1182.855244
TS-AXPL6	-839.6322628	-839.386165	TS-AXPL6	-1068.670012	-1068.350085	TS-AXPL6	-1183.201434	-1182.845077
TS-AXPL7	-839.6496681	-839.404028	TS-AXPL7	-1068.687599	-1068.367308	TS-AXPL7	-1183.221644	-1182.865119
Glucuronoxylan	-1296.659504	-1296.283781						
TS-acyclic-product	-1296.579116	-1296.209833						
TS-FFL1	-1296.537107	-1296.166575						
TS-FFL2	-1296.545061	-1296.173749						
TS-AXPL1	-1296.54563	-1296.178231						
TS-AXPL2	-1296.549606	-1296.181991						
TS-AXPL3	-1296.540718	-1296.17158						
TS-AXPL4	-1296.535164	-1296.167277						
TS-AXPL5	-1296.537248	-1296.169066						
TS-AXPL6	-1296.521073	-1296.153563						
TS-AXPL7	-1296.539049	-1296.17127						

2. Table S2. $\mathcal{T}1$ diagnostic.

**Species**	$\mathcal{T}_1$	**Species**	$\mathcal{T}_1$	**Species**	$\mathcal{T}_1$
Xylose	0.011	2-O-acetyl-xylose	0.013	4-O-methyl-D-glucuronic acid	0.013
DXP	0.012	OADXP	0.014	DGLA	0.014
TS-DXP	0.012	TS-OADXP	0.014	TS-DGLA	0.015
TS-FF1	0.016	TS-FFL1	-	TS-FFL1	0.016
TS-FF2	0.016	TS-FFL2	0.015	TS-FFL2	0.016
TS-AXP1	0.016	TS-AXPL1	0.014	TS-AXPL1	0.017
TS-AXP2	0.014	TS-AXPL2	0.016	TS-AXPL2	0.015
TS-AXP3	0.014	TS-AXPL3	0.016	TS-AXPL3	0.014
TS-AXP4	0.014	TS-AXPL4	0.014	TS-AXPL4	0.014
TS-AXP5	0.014	TS-AXPL5	0.014	TS-AXPL5	0.014
TS-AXP6	0.015	TS-AXPL6	0.015	TS-AXPL6	0.015
TS-AXP7	0.016	TS-AXPL7	0.015	TS-AXPL7	0.014
2-Methoxy-xylose	0.011	3-Methoxy-xylose	0.011	4-Methoxy-xylose	0.011
TS-acyclic-product	0.013	TS-acyclic-product	0.013	TS-acyclic-product	0.013
TS-FFL1	-	TS-FFL1	0.016	TS-FFL1	0.016
TS-FFL2	0.016	TS-FFL2	0.015	TS-FFL2	0.016
TS-AXPL1	0.016	TS-AXPL1	0.016	TS-AXPL1	0.016
TS-AXPL2	0.014	TS-AXPL2	0.014	TS-AXPL2	0.014
TS-AXPL3	0.013	TS-AXPL3	0.013	TS-AXPL3	0.014
TS-AXPL4	0.014	TS-AXPL4	0.014	TS-AXPL4	0.014
TS-AXPL5	0.014	TS-AXPL5	0.014	TS-AXPL5	0.014
TS-AXPL6	0.014	TS-AXPL6	0.014	TS-AXPL6	0.015
TS-AXPL7	0.014	TS-AXPL7	0.014	TS-AXPL7	0.014
5-Methoxy-xylose	0.011	5-Carboxy-xylose	0.012	Glucose	0.011
TS-acyclic-product	0.013	TS-acyclic-product	0.015	TS-acyclic-product	0.013
TS-FFL1	-	TS-FFL1	0.017	TS-FFL1	0.015
TS-FFL2	0.015	TS-FFL2	0.016	TS-FFL2	0.015
TS-AXPL1	0.014	TS-AXPL1	0.016	TS-AXPL1	0.016
TS-AXPL2	0.014	TS-AXPL2	0.015	TS-AXPL2	0.014
TS-AXPL3	0.014	TS-AXPL3	0.015	TS-AXPL3	0.013
TS-AXPL4	0.013	TS-AXPL4	0.014	TS-AXPL4	0.013

TS-AXPL5	0.013	TS-AXPL5	0.014	TS-AXPL5	0.014
TS-AXPL6	0.015	TS-AXPL6	0.014	TS-AXPL6	0.014
TS-AXPL7	0.014	TS-AXPL7	0.013	TS-AXPL7	0.013
3-O-acetyl-xylose	0.012	4-O-acetyl-xylose	0.012	5-O-acetyl-xylose	0.012
TS-acyclic-product	0.014	TS-acyclic-product	0.014	TS-acyclic-product	0.014
TS-FFL1	0.016	TS-FFL1	0.016	TS-FFL1	0.016
TS-FFL2	0.016	TS-FFL2	0.016	TS-FFL2	0.016
TS-AXPL1	0.016	TS-AXPL1	0.016	TS-AXPL1	0.015
TS-AXPL2	0.015	TS-AXPL2	0.015	TS-AXPL2	0.015
TS-AXPL3	0.014	TS-AXPL3	0.014	TS-AXPL3	0.014
TS-AXPL4	0.018	TS-AXPL4	0.014	TS-AXPL4	0.014
TS-AXPL5	0.018	TS-AXPL5	0.014	TS-AXPL5	0.014
TS-AXPL6	0.015	TS-AXPL6	0.017	TS-AXPL6	0.016
TS-AXPL7	0.015	TS-AXPL7	0.015	TS-AXPL7	0.014
3-Methoxy-5-methoy-xylose	0.011	3-Methoxy-5-carboxy-xylose	0.012	3-Methoxy-5-O-acetyl-xylose	0.012
TS-acyclic-product	0.013	TS-acyclic-product	0.014	TS-acyclic-product	0.014
TS-FFL1	0.016	TS-FFL1	0.017	TS-FFL1	0.016
TS-FF2	0.015	TS-FF2	0.016	TS-FF2	0.015
TS-AXPL1	0.015	TS-AXPL1	0.016	TS-AXPL1	0.014
TS-AXPL2	0.014	TS-AXPL2	0.015	TS-AXPL2	0.014
TS-AXPL3	0.013	TS-AXPL3	0.014	TS-AXPL3	0.014
TS-AXPL4	0.013	TS-AXPL4	0.014	TS-AXPL4	0.014
TS-AXPL5	0.013	TS-AXPL5	0.014	TS-AXPL5	0.014
TS-AXPL6	0.014	TS-AXPL6	0.014	TS-AXPL6	0.014
TS-AXPL7	0.014	TS-AXPL7	0.013	TS-AXPL7	0.014
3-O-acetyl-5-methoxy-xylose	0.012	3-O-acetyl-5-carboxy-xylose	0.013	3-O-acetyl-5-O-acetyl-xylose	0.013
TS-acyclic-product	0.014	TS-acyclic-product	0.015	TS-acyclic-product	0.015
TS-FFL1	0.016	TS-FFL1	0.017	TS-FFL1	0.016
TS-FFL2	0.015	TS-FFL2	0.016	TS-FFL2	0.016
TS-AXPL1	0.014	TS-AXPL1	0.016	TS-AXPL1	0.016
TS-AXPL2	0.015	TS-AXPL2	0.015	TS-AXPL2	0.015
TS-AXPL3	0.014	TS-AXPL3	0.015	TS-AXPL3	0.015
TS-AXPL4	0.016	TS-AXPL4	0.017	TS-AXPL4	0.017
TS-AXPL5	0.017	TS-AXPL5	0.017	TS-AXPL5	0.017
TS-AXPL6	0.014	TS-AXPL6	0.014	TS-AXPL6	0.014
TS-AXPL7	0.014	TS-AXPL7	0.014	TS-AXPL7	0.014
3-Methoxy-4-methoxy-5-methoxy-xylose	0.011	3-Methoxy-4-methoxy-5-carboxy-xylose	0.012	3-O-acetyl-4-methoxy-5-methoxy-xylose	0.012
TS-acyclic-product	0.013	TS-acyclic-product	0.014	TS-acyclic-product	0.014
TS-FFL1	0.015	TS-FFL1	0.017	TS-FFL1	0.016
TS-FFL2	0.014	TS-FFL2	0.015	TS-FFL2	0.015
TS-AXPL1	0.015	TS-AXPL1	0.016	TS-AXPL1	0.014
TS-AXPL2	0.014	TS-AXPL2	0.014	TS-AXPL2	0.014
TS-AXPL3	0.013	TS-AXPL3	0.014	TS-AXPL3	0.014
TS-AXPL4	0.013	TS-AXPL4	0.014	TS-AXPL4	0.016
TS-AXPL5	0.013	TS-AXPL5	0.014	TS-AXPL5	0.016
TS-AXPL6	0.014	TS-AXPL6	0.014	TS-AXPL6	0.014
TS-AXPL7	0.013	TS-AXPL7	0.013	TS-AXPL7	0.014
3-O-acetyl-4-methoxy-5-carboxy-xylose	0.013	4-Methoxy-5-methoxy-xylose	0.011	4-Methoxy-5-O-acetyl-xylose	0.012

TS-acyclic-product	0.015	TS-acyclic-product	0.013	TS-acyclic-product	0.014
TS-FFL1	0.016	TS-FFL1	-	TS-FFL1	-
TS-FFL2	0.016	TS-FFL2	0.015	TS-FFL2	0.015
TS-AXPL1	0.016	TS-AXPL1	0.015	TS-AXPL1	0.014
TS-AXPL2	0.015	TS-AXPL2	0.014	TS-AXPL2	0.014
TS-AXPL3	0.014	TS-AXPL3	0.014	TS-AXPL3	0.014
TS-AXPL4	0.017	TS-AXPL4	0.014	TS-AXPL4	0.014
TS-AXPL5	0.017	TS-AXPL5	0.013	TS-AXPL5	0.014
TS-AXPL6	0.014	TS-AXPL6	0.014	TS-AXPL6	0.015
TS-AXPL7	0.014	TS-AXPL7	0.013	TS-AXPL7	0.014
4-O-acetyl-5-methoxy-xylose	0.012	Xylobiose	0.011	Xyloglucan	0.011
TS-acyclic-product	0.014	TS-acyclic-product	0.012	TS-acyclic-product	0.012
TS-FFL1	0.016	TS-FFL1	0.014	TS-FFL1	0.014
TS-FFL2	0.015	TS-FFL2	0.014	TS-FFL2	0.014
TS-AXPL1	0.014	TS-AXPL1	0.014	TS-AXPL1	0.013
TS-AXPL2	0.014	TS-AXPL2	0.013	TS-AXPL2	0.013
TS-AXPL3	0.014	TS-AXPL3	0.013	TS-AXPL3	0.013
TS-AXPL4	0.014	TS-AXPL4	0.013	TS-AXPL4	0.012
TS-AXPL5	0.014	TS-AXPL5	0.012	TS-AXPL5	0.012
TS-AXPL6	0.016	TS-AXPL6	0.015	TS-AXPL6	0.014
TS-AXPL7	0.015	TS-AXPL7	0.014	TS-AXPL7	0.014
Glucuronoxylan	0.012				
TS-acyclic-product	0.013				
TS-FFL1	0.014				
TS-FFL2	0.014				
TS-AXPL1	0.014				
TS-AXPL2	0.014				
TS-AXPL3	0.013				
TS-AXPL4	0.013				
TS-AXPL5	0.013				
TS-AXPL6	0.015				
TS-AXPL7	0.016				

3. Table S3. Activation enthalpies, $\Delta H^{\ddagger}$, in kcal mol^{-1} at 0 and 298 K for ring-opening, ring contraction and elimination at different levels of theory.

Elementary Reaction	T = 0 K				T = 298 K			
	$\Delta H^{\ddagger}_{DFT}$	$\Delta H^{\ddagger}_{CBS}$	$\Delta H^{\ddagger}_{G4}$	$\Delta H^{\ddagger}_{DLPNO}$	$\Delta H^{\ddagger}_{DFT}$	$\Delta H^{\ddagger}_{CBS}$	$\Delta H^{\ddagger}_{G4}$	$\Delta H^{\ddagger}_{DLPNO}$
2-Methoxy-xylose ⟶ acyclic product	49.99	45.81	45.50	49.38	45.98	45.67	45.35	45.49
2-Methoxy-xylose ⟶ FFL2 + CH_3OH	70.74	63.36	62.69	66.05	68.38	63.44	62.80	63.74
2-Methoxy-xylose ⟶ AXPL1 + H_2O	74.64	68.39	68.37	73.48	69.70	68.85	68.85	68.71
2-Methoxy-xylose ⟶ AXPL2 + CH_3OH	84.20	77.94	77.66	84.37	80.04	78.46	78.23	80.34
2-Methoxy-xylose ⟶ AXPL3 + CH_3OH	76.98	72.18	71.51	76.82	72.48	72.65	71.96	72.47
2-Methoxy-xylose ⟶ AXPL4 + H_2O	80.17	74.41	73.66	79.89	74.88	74.86	73.96	74.73
2-Methoxy-xylose ⟶ AXPL5 + H_2O	80.55	74.14	74.07	80.41	75.20	74.60	74.45	75.18
2-Methoxy-xylose ⟶ AXPL6 + H_2O	76.66	71.02	70.55	76.39	71.48	71.25	70.74	71.36
2-Methoxy-xylose ⟶ AXPL7 + H_2O	74.77	70.55	69.95	77.31	69.66	70.62	69.97	72.08
3-Methoxy-xylose ⟶ acyclic product	48.69	45.11	44.72	48.42	44.69	45.09	44.68	44.54
3-Methoxy-xylose ⟶ FFL1 + H_2O	76.63	71.29	71.30	75.24	73.08	71.30	71.30	71.76
3-Methoxy-xylose ⟶ FFL2 + H_2O	72.50	64.67	64.37	68.21	69.69	64.74	64.44	65.44
3-Methoxy-xylose ⟶ AXPL1 + H_2O	73.01	67.75	67.65	72.08	68.24	68.24	68.07	67.48
3-Methoxy-xylose ⟶ AXPL2 + H_2O	87.29	80.71	80.25	87.43	81.94	81.23	80.68	82.29
3-Methoxy-xylose ⟶ AXPL3 + H_2O	78.87	73.25	72.74	78.72	73.79	73.57	73.00	73.81
3-Methoxy-xylose ⟶ AXPL4 + CH_3OH	78.28	71.05	71.35	78.22	73.39	71.64	71.98	73.47
3-Methoxy-xylose ⟶ AXPL5 + CH_3OH	76.95	70.95	70.51	77.63	72.24	71.53	71.15	73.10
3-Methoxy-xylose ⟶ AXPL6 + H_2O	79.19	71.47	71.25	80.04	73.65	71.99	71.80	74.63
3-Methoxy-xylose ⟶ AXPL7 + H_2O	75.49	70.53	69.96	75.80	70.07	70.66	70.06	70.58
4-Methoxy-xylose ⟶ acyclic product	49.45	45.85	45.44	49.23	45.37	45.84	45.40	45.24
4-Methoxy-xylose ⟶ FFL1 + H_2O	77.28	56.24	72.38	76.00	73.96	56.54	72.43	72.77
4-Methoxy-xylose ⟶ FFL2 + H_2O	72.96	65.05	64.82	68.45	70.11	65.08	64.82	65.64
4-Methoxy-xylose ⟶ AXPL1 + H_2O	75.72	69.64	69.20	74.83	70.73	70.18	69.73	69.99
4-Methoxy-xylose ⟶ AXPL2 + H_2O	87.24	81.40	80.66	87.44	82.11	81.90	81.01	82.45
4-Methoxy-xylose ⟶ AXPL3 + H_2O	79.90	74.03	73.49	79.60	74.70	74.39	73.78	74.55
4-Methoxy-xylose ⟶ AXPL4 + H_2O	81.32	75.48	74.70	81.06	76.00	75.98	75.03	75.90
4-Methoxy-xylose ⟶ AXPL5 + H_2O	80.38	74.89	74.07	80.60	75.04	75.35	74.42	75.40
4-Methoxy-xylose ⟶ AXPL6 + CH_3OH	81.21	73.13	72.95	80.19	75.73	73.69	73.52	74.76
4-Methoxy-xylose ⟶ AXPL7 + CH_3OH	76.49	71.91	71.19	76.53	71.52	72.27	71.52	71.73

DFT stands for M06-2X/6-311++G(d,p).
CBS stands for CBS-QB3.
DLPNO stands for DLPNO-CCSD(T)-F12/cc-pVTZ-F12//M06-2X/6-311++G(d,p).

4. Table S4. Activation enthalpies, $\Delta H^{\ddagger}$, in kcal mol^{-1} at 0 and 298 K for ring-opening, ring contraction and elimination at different levels of theory.

Elementary Reaction	T = 0 K				T = 298 K			
	$\Delta H^{\ddagger}_{DFT}$	$\Delta H^{\ddagger}_{CBS}$	$\Delta H^{\ddagger}_{G4}$	$\Delta H^{\ddagger}_{DLPNO}$	$\Delta H^{\ddagger}_{DFT}$	$\Delta H^{\ddagger}_{CBS}$	$\Delta H^{\ddagger}_{G4}$	$\Delta H^{\ddagger}_{DLPNO}$
5-Methoxy-xylose ⟶ acyclic product	49.27	45.67	45.18	48.88	45.03	45.68	45.17	44.77
5-Methoxy-xylose ⟶ FFL2 + H_2O	72.04	64.74	64.46	68.16	69.23	64.85	64.55	65.48
5-Methoxy-xylose ⟶ AXPL1 + H_2O	76.62	70.37	70.29	75.86	71.41	70.86	70.73	70.82
5-Methoxy-xylose ⟶ AXPL2 + H_2O	86.56	81.36	80.63	86.94	81.77	81.84	80.96	82.30
5-Methoxy-xylose ⟶ AXPL3 + H_2O	80.40	74.43	73.85	79.97	75.21	74.80	74.14	74.96
5-Methoxy-xylose ⟶ AXPL4 + H_2O	80.33	74.85	74.18	80.23	75.20	75.20	74.41	75.28
5-Methoxy-xylose ⟶ AXPL5 + H_2O	80.55	74.88	74.24	80.41	75.31	75.27	74.51	75.37
5-Methoxy-xylose ⟶ AXPL6 + H_2O	85.32	77.85	77.52	84.16	79.55	78.25	77.99	78.58
5-Methoxy-xylose ⟶ AXPL7 + H_2O	84.01	78.12	77.87	84.26	78.67	78.38	78.07	79.08
5-Carboxy-xylose ⟶ acyclic product	52.18	48.85	48.28	51.76	47.67	48.82	48.24	47.38
5-Carboxy-xylose ⟶ FFL1 + H_2O	85.48	79.36	79.44	83.36	81.86	79.54	79.54	79.84
5-Carboxy-xylose ⟶ FFL2 + H_2O	66.85	60.67	60.71	63.81	64.10	60.79	60.82	61.15
5-Carboxy-xylose ⟶ AXPL1 + H_2O	78.64	73.22	72.43	78.80	73.46	73.76	72.99	73.80
5-Carboxy-xylose ⟶ AXPL2 + H_2O	85.24	78.86	78.04	85.53	81.22	79.07	78.10	81.64
5-Carboxy-xylose ⟶ AXPL3 + H_2O	81.20	75.32	74.74	80.90	76.05	75.68	75.03	75.92
5-Carboxy-xylose ⟶ AXPL4 + H_2O	78.09	74.21	73.50	79.35	73.43	74.37	73.56	74.82
5-Carboxy-xylose ⟶ AXPL5 + H_2O	81.73	76.56	76.02	82.43	76.69	77.08	76.34	77.55
5-Carboxy-xylose ⟶ AXPL6 + H_2O	85.08	78.70	78.49	84.80	80.98	79.28	78.67	80.83
5-Carboxy-xylose ⟶ AXPL7 + H_2O	70.08	67.07	66.49	68.91	65.79	67.26	66.68	65.19
Glucose ⟶ acyclic product	78.37	72.52	71.95	76.76	73.38	73.00	72.44	71.94
Glucose ⟶ FFL1 + H_2O	90.58	79.78	79.29	91.50	85.09	79.79	79.26	86.34
Glucose ⟶ FFL2 + H_2O	83.90	77.98	77.36	83.54	78.60	78.33	77.65	78.45
Glucose ⟶ AXPL1 + H_2O	82.22	76.17	75.49	83.32	77.01	76.58	75.85	78.36
Glucose ⟶ AXPL2 + H_2O	83.20	76.59	76.32	83.06	77.70	77.13	76.71	77.77
Glucose ⟶ AXPL3 + H_2O	80.77	74.42	73.83	79.92	75.53	74.74	74.09	74.85
Glucose ⟶ AXPL4 + H_2O	78.59	73.92	73.54	78.96	73.69	73.94	73.50	74.24
Glucose ⟶ AXPL5 + H_2O	78.37	72.52	71.95	76.76	73.38	73.00	72.44	71.94
Glucose ⟶ AXPL6 + H_2O	90.58	79.78	79.29	91.50	85.09	79.79	79.26	86.34
Glucose ⟶ AXPL7 + H_2O	83.90	77.98	77.36	83.54	78.60	78.33	77.65	78.45

DFT stands for M06-2X/6-311++G(d,p).
CBS stands for CBS-QB3.
DLPNO stands for DLPNO-CCSD(T)-F12/cc-pVTZ-F12//M06-2X/6-311++G(d,p).

5. Table S5. Activation enthalpies, $\Delta H^{\ddagger}$, in kcal mol^{-1} at 0 and 298 K for ring-opening, ring contraction and elimination at different levels of theory.

Elementary Reaction	T = 0 K				T = 298 K			
	$\Delta H^{\ddagger}_{DFT}$	$\Delta H^{\ddagger}_{CBS}$	$\Delta H^{\ddagger}_{G4}$	$\Delta H^{\ddagger}_{DLPNO}$	$\Delta H^{\ddagger}_{DFT}$	$\Delta H^{\ddagger}_{CBS}$	$\Delta H^{\ddagger}_{G4}$	$\Delta H^{\ddagger}_{DLPNO}$
3-O-acetyl-xylose ⟶ acyclic product	49.47	45.69	45.33	48.99	45.45	45.60	45.21	45.13
3-O-acetyl-xylose ⟶ FFL1 + H_2O	78.21	72.89	73.10	76.78	74.68	72.87	73.05	73.41
3-O-acetyl-xylose ⟶ FFL2 + H_2O	73.58	65.59	65.52	68.67	70.71	65.55	65.53	65.94
3-O-acetyl-xylose ⟶ AXPL1 + H_2O	74.75	68.80	68.68	73.88	69.76	69.27	69.09	69.11
3-O-acetyl-xylose ⟶ AXPL2 + H_2O	87.16	81.08	80.34	87.17	81.87	81.51	80.63	82.04
3-O-acetyl-xylose ⟶ AXPL3 + H_2O	76.69	72.09	71.74	76.83	71.96	72.20	71.81	72.32
3-O-acetyl-xylose ⟶ AXPL4 + CH_3COOH	72.22	64.90	64.49	70.89	67.07	65.52	65.13	65.82
3-Methoxy-xylose ⟶ AXPL5 + CH_3COOH	71.14	64.17	63.80	69.50	65.90	64.84	64.49	64.41
3-O-acetyl-xylose ⟶ AXPL6 + H_2O	76.33	71.44	70.85	76.58	71.14	71.64	70.91	71.56
3-O-acetyl-xylose ⟶ AXPL7 + H_2O	76.17	71.18	70.69	76.00	70.84	71.13	70.58	70.90
4-O-acetyl-xylose ⟶ acyclic product	49.61	45.97	45.57	49.34	45.52	45.94	45.52	45.36
4-O-acetyl-xylose ⟶ FFL1 + H_2O	77.94	72.51	72.73	76.19	74.58	72.60	72.72	72.94
4-O-acetyl-xylose ⟶ FFL2 + H_2O	73.74	65.74	65.46	69.12	70.87	65.80	65.49	66.30
4-O-acetyl-xylose ⟶ AXPL1 + H_2O	76.11	69.85	69.91	72.35	71.00	70.39	70.43	67.66
4-O-acetyl-xylose ⟶ AXPL2 + H_2O	87.64	81.63	80.90	87.84	82.48	82.20	81.28	82.77
4-O-acetyl-xylose ⟶ AXPL3 + H_2O	78.57	73.19	72.67	78.52	73.63	73.52	72.93	73.74
4-O-acetyl-xylose ⟶ AXPL4 + H_2O	80.14	72.78	73.82	76.92	74.91	72.83	74.10	72.78
4-O-acetyl-xylose ⟶ AXPL5 + H_2O	78.90	73.99	73.44	79.62	73.83	74.28	73.66	74.71
4-O-acetyl-xylose ⟶ AXPL6 + CH_3COOH	76.25	70.08	69.19	75.06	70.76	70.65	69.71	69.77
4-O-acetyl-xylose ⟶ AXPL7 + CH_3COOH	66.79	61.90	61.85	66.54	61.74	61.87	61.76	61.64
5-O-acetyl-xylose ⟶ acyclic product	49.21	45.90	45.22	48.98	44.84	45.87	45.20	44.76
5-O-acetyl-xylose ⟶ FFL2 + H_2O	69.05	62.71	62.54	66.00	66.27	62.83	62.65	63.31
5-O-acetyl-xylose ⟶ AXPL1 + H_2O	77.44	71.33	71.35	77.78	72.21	71.79	71.75	72.72
5-O-acetyl-xylose ⟶ AXPL2 + H_2O	85.32	80.89	80.18	85.72	81.00	81.30	80.47	81.52
5-O-acetyl-xylose ⟶ AXPL3 + H_2O	80.08	72.14	73.78	79.82	74.93	72.40	74.06	74.82
5-O-acetyl-xylose ⟶ AXPL4 + H_2O	80.07	74.90	74.22	80.19	75.01	75.25	74.47	75.31
5-O-acetyl-xylose ⟶ AXPL5 + H_2O	79.74	74.64	74.07	79.91	74.80	74.97	74.30	75.12
5-O-acetyl-xylose ⟶ AXPL6 + H_2O	86.06	78.18	77.60	84.77	80.26	78.59	78.09	79.24
5-O-acetyl-xylose ⟶ AXPL7 + H_2O	80.18	75.22	75.11	81.03	75.27	75.37	75.24	76.28

DFT stands for M06-2X/6-311++G(d,p).
CBS stands for CBS-QB3.
DLPNO stands for DLPNO-CCSD(T)-F12/cc-pVTZ-F12//M06-2X/6-311++G(d,p).

6. Table S6. Activation enthalpies, $\Delta H^{\ddagger}$, in kcal mol^{-1} at 0 and 298 K for ring-opening, ring contraction and elimination at different levels of theory.

Elementary Reaction	T = 0 K				T = 298 K			
	$\Delta H^{\ddagger}_{DFT}$	$\Delta H^{\ddagger}_{CBS}$	$\Delta H^{\ddagger}_{G4}$	$\Delta H^{\ddagger}_{DLPNO}$	$\Delta H^{\ddagger}_{DFT}$	$\Delta H^{\ddagger}_{CBS}$	$\Delta H^{\ddagger}_{G4}$	$\Delta H^{\ddagger}_{DLPNO}$
3-Methoxy-5-methoxy-xylose ⟶ acyclic product	50.28	46.51	45.98	49.76	46.09	46.52	45.99	45.70
3-Methoxy-5-methoxy-xylose ⟶ FFL1 + H_2O	79.44	73.42	73.17	77.50	75.52	73.58	73.30	73.71
3-Methoxy-5-methoxy-xylose ⟶ FFL2 + H_2O	71.19	64.08	63.72	67.52	68.45	64.19	63.83	64.88
3-Methoxy-5-methoxy-xylose ⟶ AXPL1 + H_2O	76.50	70.21	69.98	75.74	71.25	70.77	70.47	70.67
3-Methoxy-5-methoxy-xylose ⟶ AXPL2 + H_2O	86.62	80.48	80.04	87.00	81.49	80.96	80.45	82.03
3-Methoxy-5-methoxy-xylose ⟶ AXPL3 + H_2O	79.68	73.80	73.19	79.34	74.45	74.16	73.49	74.28
3-Methoxy-5-methoxy-xylose ⟶ AXPL4 + CH_3OH	77.20	71.57	70.94	77.39	72.50	72.14	71.52	72.84
3-Methoxy-5-methoxy-xylose ⟶ AXPL5 + CH_3OH	77.43	71.56	70.98	77.57	72.62	72.16	71.58	72.91
3-Methoxy-5-methoxy-xylose ⟶ AXPL6 + H_2O	83.95	77.16	76.44	83.38	78.23	77.56	76.91	77.84
3-Methoxy-5-methoxy-xylose ⟶ AXPL7 + H_2O	83.23	77.39	76.92	83.68	77.76	77.74	77.22	78.38
3-Methoxy-5-carboxy-xylose ⟶ acyclic product	53.59	49.62	48.95	53.06	49.07	49.66	49.00	48.67
3-Methoxy-5-carboxy-xylose ⟶ FFL1 + H_2O	85.45	78.91	78.68	83.26	81.44	79.12	78.86	79.38
3-Methoxy-5-carboxy-xylose ⟶ FFL2 + H_2O	66.05	59.97	59.94	63.08	63.35	60.08	60.05	60.46
3-Methoxy-5-carboxy-xylose ⟶ AXPL1 + H_2O	78.43	73.00	72.23	78.57	73.21	73.57	72.85	73.52
3-Methoxy-5-carboxy-xylose ⟶ AXPL2 + H_2O	85.52	81.12	80.38	85.82	81.33	81.58	80.74	81.76
3-Methoxy-5-carboxy-xylose ⟶ AXPL3 + H_2O	80.21	74.43	73.90	80.00	75.08	74.80	74.19	75.04
3-Methoxy-5-carboxy-xylose ⟶ AXPL4 + CH_3OH	77.23	71.41	71.36	79.13	72.33	71.96	71.91	74.39
3-Methoxy-5-carboxy-xylose ⟶ AXPL5 + CH_3OH	78.18	72.16	71.50	79.32	73.61	72.69	72.01	74.90
3-Methoxy-5-carboxy-xylose ⟶ AXPL6 + H_2O	83.68	77.49	76.78	83.65	79.39	78.05	77.46	79.49
3-Methoxy-5-carboxy-xylose ⟶ AXPL7 + H_2O	69.04	66.48	65.81	70.57	64.98	66.45	65.78	66.63
3-Methoxy-5-O-acetyl-xylose ⟶ acyclic product	50.27	46.69	46.08	49.95	46.01	46.66	46.07	45.81
3-Methoxy-5-O-acetyl-xylose ⟶ FFL1 + H_2O	81.74	75.59	75.40	79.62	78.02	75.70	75.50	76.01
3-Methoxy-5-O-acetyl-xylose ⟶ FFL2 + H_2O	68.34	62.16	61.92	65.38	65.67	62.28	62.04	62.79
3-Methoxy-5-O-acetyl-xylose ⟶ AXPL1 + H_2O	77.19	71.17	70.97	77.44	71.92	71.69	71.42	72.34
3-Methoxy-5-O-acetyl-xylose ⟶ AXPL2 + H_2O	85.44	80.04	79.70	85.88	80.96	80.50	80.09	81.53
3-Methoxy-5-O-acetyl-xylose ⟶ AXPL3 + H_2O	79.37	73.73	73.18	79.19	74.33	74.07	73.47	74.30
3-Methoxy-5-O-acetyl-xylose ⟶ AXPL4 + CH_3OH	76.68	71.62	71.03	77.14	72.15	72.18	71.59	72.75
3-Methoxy-5-O-acetyl-xylose ⟶ AXPL5 + CH_3OH	76.26	71.42	70.93	76.82	71.84	71.97	71.49	72.53
3-Methoxy-5-O-acetyl-xylose ⟶ AXPL6 + H_2O	80.16	74.36	73.81	79.77	75.09	74.67	74.05	74.85
3-Methoxy-5-O-acetyl-xylose ⟶ AXPL7 + H_2O	79.38	74.42	74.12	80.36	74.34	74.65	74.32	75.47

DFT stands for M06-2X/6-311++G(d,p).
CBS stands for CBS-QB3.
DLPNO stands for DLPNO-CCSD(T)-F12/cc-pVTZ-F12//M06-2X/6-311++G(d,p).

7. Table S7. Activation enthalpies, $\Delta H^{\ddagger}$, in kcal mol^{-1} at 0 and 298 K for ring-opening, ring contraction and elimination at different levels of theory.

Elementary Reaction	T = 0 K				T = 298 K			
	$\Delta H^{\ddagger}_{DFT}$	$\Delta H^{\ddagger}_{CBS}$	$\Delta H^{\ddagger}_{G4}$	$\Delta H^{\ddagger}_{DLPNO}$	$\Delta H^{\ddagger}_{DFT}$	$\Delta H^{\ddagger}_{CBS}$	$\Delta H^{\ddagger}_{G4}$	$\Delta H^{\ddagger}_{DLPNO}$
3-O-acetyl-5-methoxy-xylose ⟶ acyclic product	50.49	46.73	46.32	50.04	46.26	46.71	46.28	45.88
3-O-acetyl-5-methoxy-xylose ⟶ FFL1 + H_2O	80.37	74.37	74.31	78.55	76.60	74.56	74.45	74.84
3-O-acetyl-5-methoxy-xylose ⟶ FFL2 + H_2O	72.39	64.98	64.67	68.07	69.72	65.00	64.69	65.41
3-O-acetyl-5-methoxy-xylose ⟶ AXPL1 + H_2O	76.04	70.14	69.97	76.23	70.97	70.65	70.35	71.25
3-O-acetyl-5-methoxy-xylose ⟶ AXPL2 + H_2O	86.61	81.11	80.24	86.84	81.69	81.49	80.55	81.95
3-O-acetyl-5-methoxy-xylose ⟶ AXPL3 + H_2O	77.28	72.42	71.96	77.38	72.59	72.59	72.10	72.75
3-O-acetyl-5-methoxy-xylose ⟶ AXPL4 + CH_3COOH	72.76	64.95	64.66	70.64	67.57	65.60	65.35	65.52
3-O-acetyl-5-methoxy-xylose ⟶ AXPL5 + CH_3COOH	72.51	64.87	64.61	70.64	67.28	65.54	65.30	65.51
3-O-acetyl-5-methoxy-xylose ⟶ AXPL6 + H_2O	79.40	74.25	73.63	79.41	74.47	74.50	73.77	74.55
3-O-acetyl-5-methoxy-xylose ⟶ AXPL7 + H_2O	83.53	77.71	77.26	83.77	78.23	77.93	77.43	78.58
3-O-acetyl-5-carboxy-xylose ⟶ acyclic product	53.64	49.75	48.74	53.98	49.07	49.76	49.06	49.68
3-O-acetyl-5-carboxy-xylose ⟶ FFL1 + H_2O	88.43	81.88	81.57	86.58	84.61	82.28	81.96	82.90
3-O-acetyl-5-carboxy-xylose ⟶ FFL2 + H_2O	67.48	60.91	60.86	63.89	64.81	60.92	60.88	61.31
3-O-acetyl-5-carboxy-xylose ⟶ AXPL1 + H_2O	77.68	72.53	71.85	78.06	72.53	73.02	72.34	73.13
3-O-acetyl-5-carboxy-xylose ⟶ AXPL2 + H_2O	85.36	81.04	80.28	85.54	81.21	81.39	80.55	81.53
3-O-acetyl-5-carboxy-xylose ⟶ AXPL3 + H_2O	77.90	73.09	72.68	78.09	73.18	73.27	72.83	73.54
3-O-acetyl-5-carboxy-xylose ⟶ AXPL4 + CH_3COOH	73.66	65.91	65.66	71.62	68.47	66.56	66.34	66.62
3-O-acetyl-5-carboxy-xylose ⟶ AXPL5 + CH_3COOH	73.71	66.09	65.91	72.00	68.56	66.74	66.60	67.01
3-O-acetyl-5-carboxy-xylose ⟶ AXPL6 + H_2O	78.32	73.64	73.98	78.63	73.47	74.00	73.98	74.94
3-O-acetyl-5-carboxy-xylose ⟶ AXPL7 + H_2O	76.41	69.33	68.60	77.72	72.27	69.35	68.62	73.71
3-O-acetyl-5-O-acetyl-xylose ⟶ acyclic product	50.49	46.90	46.42	50.28	46.11	46.85	46.36	46.03
3-O-acetyl-5-O-acetyl-xylose ⟶ FFL1 + H_2O	82.69	76.64	76.64	80.82	79.06	76.78	76.75	77.31
3-O-acetyl-5-O-acetyl-xylose ⟶ FFL2 + H_2O	69.82	63.12	62.93	66.11	67.10	63.14	62.96	63.48
3-O-acetyl-5-O-acetyl-xylose ⟶ AXPL1 + H_2O	76.50	71.18	70.88	77.16	71.45	71.67	71.22	72.26
3-O-acetyl-5-O-acetyl-xylose ⟶ AXPL2 + H_2O	85.51	80.68	79.89	85.81	81.06	81.03	80.17	81.50
3-O-acetyl-5-O-acetyl-xylose ⟶ AXPL3 + H_2O	77.04	72.45	72.02	77.28	72.45	72.60	72.14	72.83
3-O-acetyl-5-O-acetyl-xylose ⟶ AXPL4 + CH_3COOH	73.21	65.55	65.35	71.46	67.95	66.19	66.02	66.39
3-O-acetyl-5-O-acetyl-xylose ⟶ AXPL5 + CH_3COOH	73.57	65.82	65.72	71.88	68.33	66.47	66.38	66.81
3-O-acetyl-5-O-acetyl-xylose ⟶ AXPL6 + H_2O	81.01	73.11	74.99	81.00	75.91	73.24	75.18	76.06
3-O-acetyl-5-O-acetyl-xylose ⟶ AXPL7 + H_2O	79.71	74.89	74.65	80.50	74.83	75.02	74.75	75.77

DFT stands for M06-2X/6-311++G(d,p).
CBS stands for CBS-QB3.
DLPNO stands for DLPNO-CCSD(T)-F12/cc-pVTZ-F12//M06-2X/6-311++G(d,p).

8. Table S8. Activation enthalpies, $\Delta H^{\ddagger}$, in kcal mol^{-1} at 0 and 298 K for ring-opening, ring contraction and elimination at different levels of theory.

Elementary Reaction	T = 0 K				T = 298 K			
	$\Delta H^{\ddagger}_{DFT}$	$\Delta H^{\ddagger}_{CBS}$	$\Delta H^{\ddagger}_{G4}$	$\Delta H^{\ddagger}_{DLPNO}$	$\Delta H^{\ddagger}_{DFT}$	$\Delta H^{\ddagger}_{CBS}$	$\Delta H^{\ddagger}_{G4}$	$\Delta H^{\ddagger}_{DLPNO}$
3-Methoxy-4-methoxy-5-methoxy-xylose ⟶ acyclic product	49.58	45.73	45.27	49.11	45.47	45.70	45.21	45.12
3-Methoxy-4-methoxy-5-methoxy-xylose ⟶ FFL1 + H_2O	78.95	72.99	72.83	77.04	75.13	73.12	72.93	73.34
3-Methoxy-4-methoxy-5-methoxy-xylose ⟶ FFL2 + H_2O	70.86	63.64	63.37	66.95	68.15	63.71	63.43	64.32
3-Methoxy-4-methoxy-5-methoxy-xylose ⟶ AXPL1 + H_2O	75.67	69.32	69.68	74.77	70.47	69.87	70.13	69.74
3-Methoxy-4-methoxy-5-methoxy-xylose ⟶ AXPL2 + H_2O	86.96	80.01	80.41	87.35	81.66	80.42	80.86	82.21
3-Methoxy-4-methoxy-5-methoxy-xylose ⟶ AXPL3 + H_2O	79.24	73.41	72.87	78.98	74.06	73.73	73.13	73.95
3-Methoxy-4-methoxy-5-methoxy-xylose ⟶ AXPL4 + CH_3OH	77.14	71.01	70.46	76.80	72.25	71.56	70.93	72.07
3-Methoxy-4-methoxy-5-methoxy-xylose ⟶ AXPL5 + CH_3OH	75.64	70.14	69.61	75.84	70.91	70.61	70.10	71.25
3-Methoxy-4-methoxy-5-methoxy-xylose ⟶ AXPL6 + CH_3OH	78.73	71.10	70.70	78.22	73.61	71.67	71.09	73.26
3-Methoxy-4-methoxy-5-methoxy-xylose ⟶ AXPL7 + CH_3OH	79.43	74.85	74.23	80.18	74.70	75.36	74.69	75.59
3-Methoxy-4-methoxy-5-carboxy-xylose ⟶ acyclic product	51.84	47.98	47.68	51.52	47.38	47.90	47.57	47.19
3-Methoxy-4-methoxy-5-carboxy-xylose ⟶ FFL1 + H_2O	82.94	76.60	76.53	80.90	79.03	76.71	76.62	77.12
3-Methoxy-4-methoxy-5-carboxy-xylose ⟶ FFL2 + H_2O	67.03	60.70	60.80	63.95	64.44	60.77	60.85	61.45
3-Methoxy-4-methoxy-5-carboxy-xylose ⟶ AXPL1 + H_2O	75.13	69.52	68.32	75.15	70.04	69.97	68.75	70.22
3-Methoxy-4-methoxy-5-carboxy-xylose ⟶ AXPL2 + H_2O	86.00	81.27	75.54	86.29	81.45	81.73	75.45	81.89
3-Methoxy-4-methoxy-5-carboxy-xylose ⟶ AXPL3 + H_2O	79.79	74.15	71.86	79.44	74.66	74.51	72.00	74.47
3-Methoxy-4-methoxy-5-carboxy-xylose ⟶ AXPL4 + CH_3OH	72.17	66.48	66.79	73.76	67.67	66.73	67.02	69.39
3-Methoxy-4-methoxy-5-carboxy-xylose ⟶ AXPL5 + CH_3OH	74.84	70.43	70.22	75.73	70.46	70.94	70.70	71.49
3-Methoxy-4-methoxy-5-carboxy-xylose ⟶ AXPL6 + CH_3OH	77.80	70.68	70.74	78.02	72.91	71.16	71.24	73.28
3-Methoxy-4-methoxy-5-carboxy-xylose ⟶ AXPL7 + CH_3OH	61.86	59.58	59.13	63.04	58.20	59.59	59.14	59.49
3-O-acetyl-4-methoxy-5-methoxy-xylose ⟶ acyclic product	49.72	45.87	45.41	49.36	45.51	45.86	45.38	45.27
3-O-acetyl-4-methoxy-5-methoxy-xylose ⟶ FFL1 + H_2O	79.97	74.03	73.95	78.08	76.28	74.22	74.09	74.48
3-O-acetyl-4-methoxy-5-methoxy-xylose ⟶ FFL2 + H_2O	72.40	64.73	64.51	67.73	69.74	64.68	64.45	65.15
3-O-acetyl-4-methoxy-5-methoxy-xylose ⟶ AXPL1 + H_2O	74.52	69.45	69.99	75.60	69.96	69.94	70.14	71.17
3-O-acetyl-4-methoxy-5-methoxy-xylose ⟶ AXPL2 + H_2O	87.02	81.49	80.55	87.21	82.04	81.89	80.85	82.37
3-O-acetyl-4-methoxy-5-methoxy-xylose ⟶ AXPL3 + H_2O	77.42	72.47	72.11	77.44	72.63	72.63	72.22	72.78
3-O-acetyl-4-methoxy-5-methoxy-xylose ⟶ AXPL4 + CH_3COOH	72.71	65.09	64.63	70.69	67.55	65.76	65.30	65.65
3-O-acetyl-4-methoxy-5-methoxy-xylose ⟶ AXPL5 + CH_3COOH	71.96	64.61	64.11	70.10	66.72	65.20	64.67	65.02
3-O-acetyl-4-methoxy-5-methoxy-xylose ⟶ AXPL6 + CH_3OH	76.57	69.99	71.16	77.05	72.34	70.22	71.56	72.86
3-O-acetyl-4-methoxy-5-methoxy-xylose ⟶ AXPL7 + CH_3OH	80.44	75.99	75.57	80.76	76.04	76.33	75.88	76.49

DFT stands for M06-2X/6-311++G(d,p).
CBS stands for CBS-QB3.
DLPNO stands for DLPNO-CCSD(T)-F12/cc-pVTZ-F12//M06-2X/6-311++G(d,p).

9. Table S9. Activation enthalpies, $\Delta H^{\ddagger}$, in kcal mol^{-1} at 0 and 298 K for ring-opening, ring contraction and elimination at different levels of theory.

Elementary Reaction	T = 0 K				T = 298 K			
	$\Delta H^{\ddagger}_{DFT}$	$\Delta H^{\ddagger}_{CBS}$	$\Delta H^{\ddagger}_{G4}$	$\Delta H^{\ddagger}_{DLPNO}$	$\Delta H^{\ddagger}_{DFT}$	$\Delta H^{\ddagger}_{CBS}$	$\Delta H^{\ddagger}_{G4}$	$\Delta H^{\ddagger}_{DLPNO}$
3-O-acetyl-4-methoxy-5-carboxy-xylose ⟶ acyclic product	51.92	47.91	47.67	51.68	47.43	47.89	47.62	47.32
3-O-acetyl-4-methoxy-5-carboxy-xylose ⟶ FFL1 + H_2O	84.34	78.17	78.17	82.35	80.65	78.32	78.29	78.77
3-O-acetyl-4-methoxy-5-carboxy-xylose ⟶ FFL2 + H_2O	68.60	61.66	61.75	64.72	65.94	61.61	61.69	62.15
3-O-acetyl-4-methoxy-5-carboxy-xylose ⟶ AXPL1 + H_2O	75.26	69.45	68.26	75.46	70.10	69.83	68.73	70.47
3-O-acetyl-4-methoxy-5-carboxy-xylose ⟶ AXPL2 + H_2O	85.80	81.24	75.52	86.03	81.30	81.60	75.36	81.67
3-O-acetyl-4-methoxy-5-carboxy-xylose ⟶ AXPL3 + H_2O	78.04	73.14	72.77	78.03	73.27	73.32	72.90	73.40
3-O-acetyl-4-methoxy-5-carboxy-xylose ⟶ AXPL4 + CH_3COOH	71.82	63.95	63.41	69.67	66.59	64.62	64.11	64.62
3-O-acetyl-4-methoxy-5-carboxy-xylose ⟶ AXPL5 + CH_3COOH	72.95	65.14	64.58	71.18	67.62	65.82	65.25	66.03
3-O-acetyl-4-methoxy-5-carboxy-xylose ⟶ AXPL6 + CH_3OH	74.03	67.48	66.96	74.54	69.69	67.66	67.14	70.33
3-O-acetyl-4-methoxy-5-carboxy-xylose ⟶ AXPL7 + CH_3OH	57.69	55.45	55.49	59.30	54.09	55.43	55.40	55.80
4-Methoxy-5-methoxy-xylose ⟶ acyclic product	48.84	45.15	44.67	48.44	44.60	45.13	44.64	44.32
4-Methoxy-5-methoxy-xylose ⟶ FFL2 + H_2O	71.77	64.32	64.07	67.73	69.01	64.39	64.12	65.07
4-Methoxy-5-methoxy-xylose ⟶ AXPL1 + H_2O	76.13	69.81	69.90	75.26	70.88	70.31	70.38	70.18
4-Methoxy-5-methoxy-xylose ⟶ AXPL2 + H_2O	87.05	81.72	80.98	87.36	82.20	82.20	81.33	82.66
4-Methoxy-5-methoxy-xylose ⟶ AXPL3 + H_2O	80.24	74.12	73.57	79.78	74.91	74.50	73.87	74.62
4-Methoxy-5-methoxy-xylose ⟶ AXPL4 + H_2O	80.83	74.61	73.77	79.99	75.45	75.03	74.09	74.79
4-Methoxy-5-methoxy-xylose ⟶ AXPL5 + H_2O	79.23	73.64	72.95	79.19	73.97	74.01	73.23	74.09
4-Methoxy-5-methoxy-xylose ⟶ AXPL6 + CH_3OH	82.94	74.57	74.44	81.76	77.38	75.17	75.05	76.39
4-Methoxy-5-methoxy-xylose ⟶ AXPL7 + CH_3OH	81.00	76.46	75.98	81.51	76.35	76.93	76.43	77.00
4-Methoxy-5-O-acetyl-xylose ⟶ acyclic product	48.83	45.43	44.86	48.57	44.52	45.36	44.79	44.39
4-Methoxy-5-O-acetyl-xylose ⟶ FFL2 + H_2O	69.12	62.57	62.41	65.96	66.37	62.67	62.48	63.31
4-Methoxy-5-O-acetyl-xylose ⟶ AXPL1 + H_2O	76.63	70.89	70.92	78.04	71.68	71.34	71.36	73.24
4-Methoxy-5-O-acetyl-xylose ⟶ AXPL2 + H_2O	85.70	81.22	80.51	86.03	81.43	81.64	80.81	81.89
4-Methoxy-5-O-acetyl-xylose ⟶ AXPL3 + H_2O	80.00	71.27	73.59	79.71	74.76	71.52	73.89	74.63
4-Methoxy-5-O-acetyl-xylose ⟶ AXPL4 + H_2O	80.52	74.96	74.20	80.37	75.30	75.39	74.54	75.31
4-Methoxy-5-O-acetyl-xylose ⟶ AXPL5 + H_2O	78.74	73.64	72.98	78.93	73.74	73.94	73.21	74.08
4-Methoxy-5-O-acetyl-xylose ⟶ AXPL6 + CH_3OH	83.92	75.31	75.27	82.70	78.36	75.92	75.86	77.32
4-Methoxy-5-O-acetyl-xylose ⟶ AXPL7 + CH_3OH	76.79	73.16	72.92	77.74	72.86	73.49	73.24	73.94

DFT stands for M06-2X/6-311++G(d,p).
CBS stands for CBS-QB3.
DLPNO stands for DLPNO-CCSD(T)-F12/cc-pVTZ-F12//M06-2X/6-311++G(d,p).

10. Table S10. Activation enthalpies, $\Delta H^{\ddagger}$, in kcal mol^{-1} at 0 and 298 K for ring-opening, ring contraction and elimination at different levels of theory.

Elementary Reaction	T = 0 K				T = 298 K			
	$\Delta H^{\ddagger}_{DFT}$	$\Delta H^{\ddagger}_{CBS}$	$\Delta H^{\ddagger}_{G4}$	$\Delta H^{\ddagger}_{DLPNO}$	$\Delta H^{\ddagger}_{DFT}$	$\Delta H^{\ddagger}_{CBS}$	$\Delta H^{\ddagger}_{G4}$	$\Delta H^{\ddagger}_{DLPNO}$
4-O-acetyl-5-methoxy-xylose ⟶ acyclic product	49.30	45.54	45.06	48.93	45.09	45.53	45.04	44.85
4-O-acetyl-5-methoxy-xylose ⟶ FFL2 + H_2O	67.52	61.25	61.20	64.02	65.00	61.04	60.95	61.57
4-O-acetyl-5-methoxy-xylose ⟶ AXPL1 + H_2O	76.13	70.89	70.01	76.41	70.95	71.51	70.45	71.40
4-O-acetyl-5-methoxy-xylose ⟶ AXPL2 + H_2O	87.05	77.96	77.69	87.41	82.16	78.17	77.78	82.67
4-O-acetyl-5-methoxy-xylose ⟶ AXPL3 + H_2O	79.35	73.66	73.17	79.12	74.29	74.02	73.45	74.21
4-O-acetyl-5-methoxy-xylose ⟶ AXPL4 + H_2O	74.48	71.26	70.82	75.13	70.39	71.30	70.78	71.16
4-O-acetyl-5-methoxy-xylose ⟶ AXPL5 + H_2O	74.75	71.45	70.88	76.45	70.19	71.60	70.97	72.03
4-O-acetyl-5-methoxy-xylose ⟶ AXPL6 + CH_3COOH	77.22	70.56	69.96	75.84	71.95	71.15	70.51	70.75
4-O-acetyl-5-methoxy-xylose ⟶ AXPL7 + CH_3COOH	68.95	65.13	64.81	69.20	64.53	65.19	64.84	64.92
Xylobiose ⟶ acyclic product	50.03	46.21	45.80	49.55	45.91	46.20	45.75	45.56
Xylobiose ⟶ FFL1 + H_2O	82.14	76.03	76.15	78.63	78.62	76.37	76.41	75.33
Xylobiose ⟶ FFL2 + H_2O	71.06	63.38	63.16	66.73	68.23	63.38	63.16	63.99
Xylobiose ⟶ AXPL1 + H_2O	75.34	69.24	68.77	74.47	70.34	69.75	69.36	69.63
Xylobiose ⟶ AXPL2 + H_2O	86.37	80.16	79.74	86.67	81.14	80.64	80.12	81.61
Xylobiose ⟶ AXPL3 + H_2O	78.20	72.51	72.09	78.07	73.12	72.80	72.32	73.15
Xylobiose ⟶ AXPL4 + H_2O	79.74	74.16	73.50	79.90	74.71	74.60	73.90	75.03
Xylobiose ⟶ AXPL5 + H_2O	74.56	71.04	70.82	75.11	70.73	71.13	70.94	71.40
Xylobiose ⟶ AXPL6 + xylose	74.10	67.36	66.96	72.79	68.83	67.71	67.30	67.69
Xylobiose ⟶ AXPL7 + xylose	75.70	69.70	69.44	77.06	70.52	69.91	69.66	71.90
Xyloglucan ⟶ acyclic product	50.97	46.60	50.97	50.26	46.55	46.82	46.55	45.98
Xyloglucan ⟶ FFL1 + H_2O	81.25	73.02	81.25	76.66	77.62	73.03	77.62	73.32
Xyloglucan ⟶ FFL2 + H_2O	69.61	62.18	69.61	65.57	66.92	62.18	66.92	62.96
Xyloglucan ⟶ AXPL1 + H_2O	73.05	68.30	73.05	73.61	68.14	68.63	68.14	68.84
Xyloglucan ⟶ AXPL2 + H_2O	85.73	79.92	85.73	86.13	80.71	80.42	80.71	81.26
Xyloglucan ⟶ AXPL3 + H_2O	79.52	73.27	79.52	78.90	74.33	73.55	74.33	73.87
Xyloglucan ⟶ AXPL4 + H_2O	80.54	74.47	80.54	80.15	75.37	74.90	75.37	75.15
Xyloglucan ⟶ AXPL5 + H_2O	75.34	71.03	75.34	75.73	71.12	71.17	71.12	71.64
Xyloglucan ⟶ AXPL6 + xylose	75.75	68.27	75.75	73.58	70.51	68.59	70.51	68.51
Xyloglucan ⟶ AXPL7 + xylose	78.24	70.04	78.24	77.05	72.79	70.57	72.79	71.78

DFT stands for M06-2X/6-311++G(d,p).
CBS stands for CBS-QB3.
DLPNO stands for DLPNO-CCSD(T)-F12/cc-pVTZ-F12//M06-2X/6-311++G(d,p).

11. Table S11. Activation enthalpies, $\Delta H^{\ddagger}$, in kcal mol^{-1} at 0 and 298 K for ring-opening, ring contraction and elimination at different levels of theory.

Elementary Reaction	T = 0 K				T = 298 K			
	$\Delta H^{\ddagger}_{DFT}$	$\Delta H^{\ddagger}_{CBS}$	$\Delta H^{\ddagger}_{G4}$	$\Delta H^{\ddagger}_{DLPNO}$	$\Delta H^{\ddagger}_{DFT}$	$\Delta H^{\ddagger}_{CBS}$	$\Delta H^{\ddagger}_{G4}$	$\Delta H^{\ddagger}_{DLPNO}$
Glucuronoxylan ⟶ acyclic product	50.44	46.49	46.12	49.84	46.40	46.48	46.08	45.92
Glucuronoxylan ⟶ FFL1 + H_2O	76.81	71.61	71.68	76.30	73.55	71.87	71.92	73.14
Glucuronoxylan ⟶ FFL2 + H_2O	71.81	63.92	63.75	67.26	69.05	63.93	63.77	64.57
Glucuronoxylan ⟶ AXPL1 + H_2O	75.59	69.71	69.03	74.76	70.60	70.22	69.58	69.94
Glucuronoxylan ⟶ AXPL2 + H_2O	86.87	80.62	80.08	86.85	81.71	81.12	80.47	81.86
Glucuronoxylan ⟶ AXPL3 + H_2O	76.72	71.81	71.39	76.94	71.98	72.07	71.60	72.35
Glucuronoxylan ⟶ AXPL4 + H_2O	78.02	70.31	69.76	78.12	73.11	70.66	70.08	73.36
Glucuronoxylan ⟶ AXPL5 + H_2O	74.54	70.54	70.31	75.08	70.41	70.77	70.53	71.07
Glucuronoxylan ⟶ AXPL6 + 4-O-methyl-D-glucuronic acid	68.96	63.15	62.63	68.62	63.87	63.42	62.91	63.68
Glucuronoxylan ⟶ AXPL7 + 4-O-methyl-D-glucuronic acid	71.46	65.11	63.89	70.30	66.23	65.45	64.29	65.24

DFT stands for M06-2X/6-311++G(d,p).
CBS stands for CBS-QB3.
DLPNO stands for DLPNO-CCSD(T)-F12/cc-pVTZ-F12//M06-2X/6-311++G(d,p).

12. Extrapolation relations for extrapolating standard activation enthalpies from B3LYP/6-31+G(d,p) to CBS-QB3, G4, and DLPNO-CCSD(T)-F12/cc-pVTZ-F12//M06-2X/6-311++G(d,p).

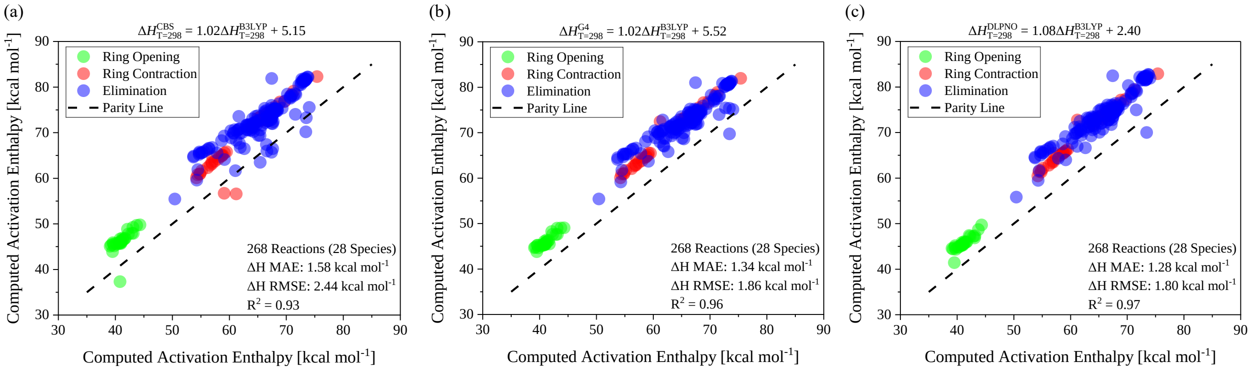

Figure S1. Parity plots of the $\Delta H^{\ddagger}_{T=298}$ MAE for extrapolating computed barriers at B3LYP/6-31+G(d,p) to CBS-QB3 (Panel a), G4 (Panel b), and DLPNO-CCSD(T)-F12/cc-pVTZ-F12//M06-2X/6-311++G(d,p) (Panel c). The reactivity indices were computed from wavefunctions at the M06-2X/6-311++G(d,p) level.

13. Extrapolation relations for extrapolating standard activation enthalpies from ωB97X-D/6-31+G(d,p) to CBS-QB3, G4, and DLPNO-CCSD(T)-F12/cc-pVTZ-F12//M06-2X/6-311++G(d,p).

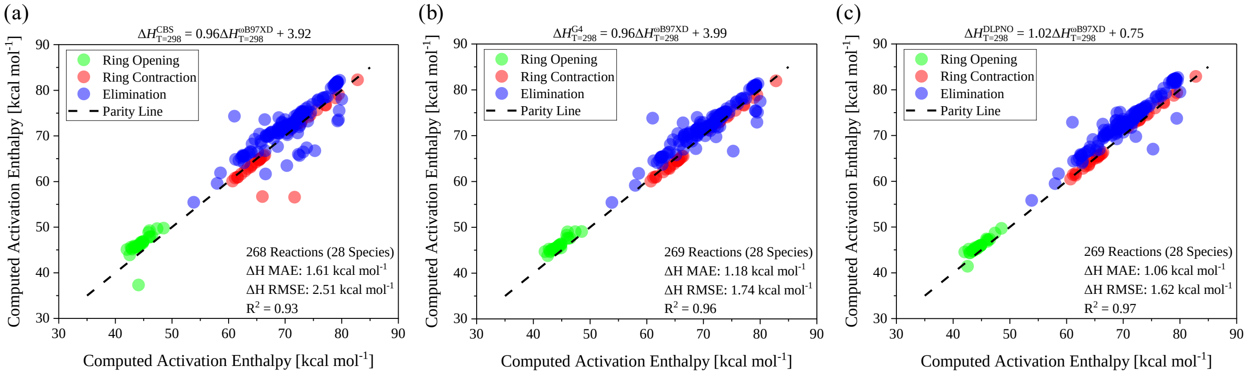

Figure S2. Parity plots of the $\Delta H^{\ddagger}_{T=298}$ MAE for extrapolating computed barriers at ωB97X-D/6-31+G(d,p) to CBS-QB3 (Panel a), G4 (Panel b), and DLPNO-CCSD(T)-F12/cc-pVTZ-F12//M06-2X/6-311++G(d,p) (Panel c). The reactivity indices were computed from wavefunctions at the M06-2X/6-311++G(d,p) level.

14. Wall-clock time required to calculate the electron population of C-O bonds across mono- and di-saccharides species.

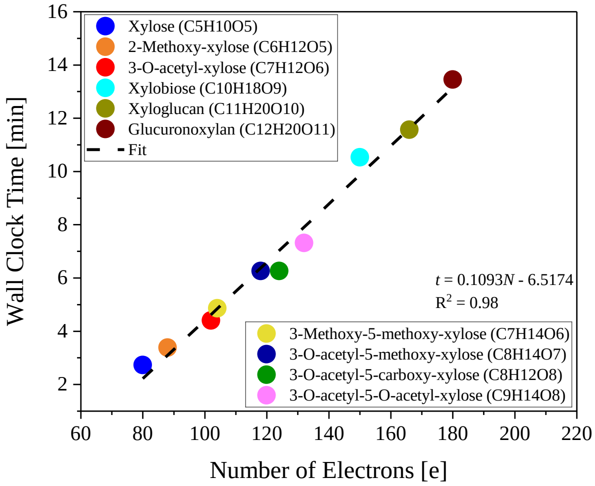

Figure S3. Wall-clock time required to compute the electron population of reactants through the topological analysis of the gradient of the ELF, showing a strong linear correlation with the size (i.e., number of electrons) of the investigated systems. The wavefunctions were generated at the M06-2X/6-311++G(d,p) level.

15. Benchmark

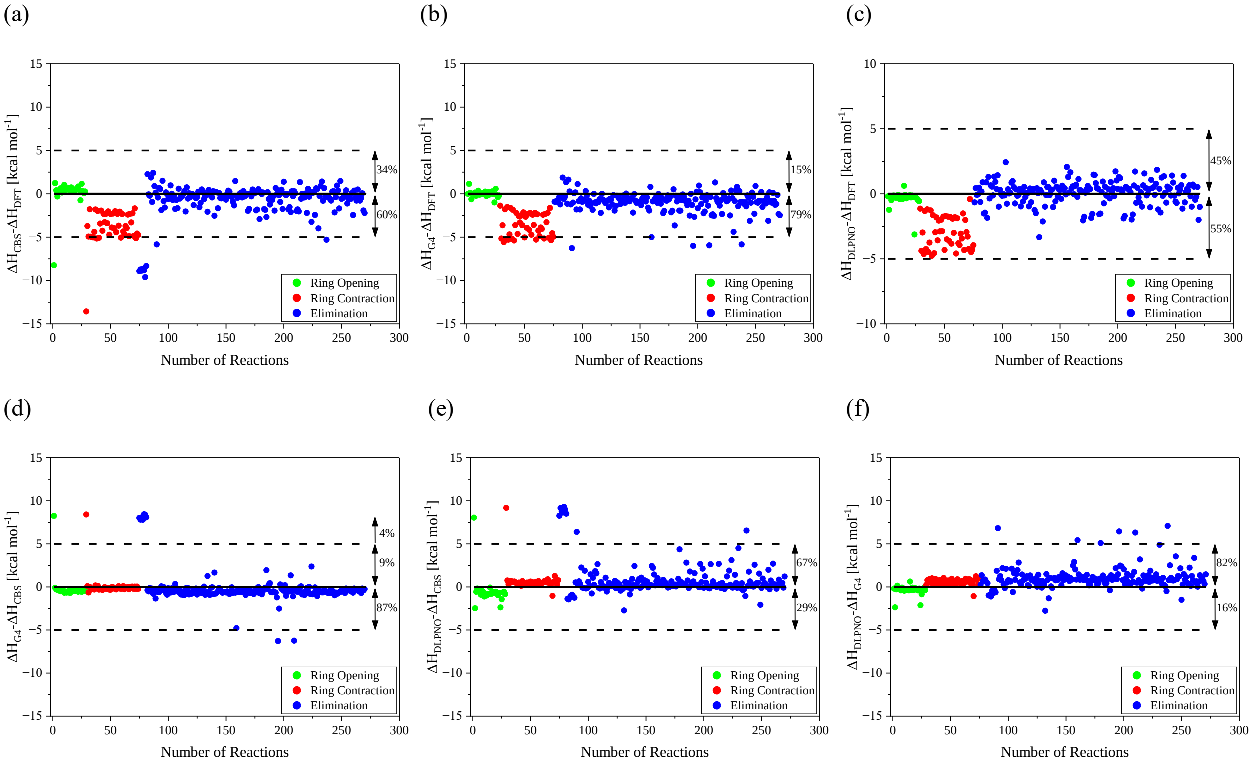

Figure S4. Assessment of barriers' significant difference (> ±5.0 kcal mol^{-1}) across several levels of theory: CBS vs DFT (Panel a), G4 vs DFT (Panel b), DLPNO vs DFT (Panel c), G4 vs CBS (Panel d), DLPNO vs CBS (Panel e), and DLPNO vs G4 (Panel f). The predicting performance of DFT in hemicellulose pyrolysis is remarkable, as shown in Panel c. CBS stands for DFT stands for M06-2X/6-311++G(d,p), CBS means CBS-QB3, and DLPNO stands for DLPNO-CCSD(T)-F12/cc-pVTZ-F12//M06-2X/6-311++G(d,p).
